# Modular analysis of the control of flagellar Ca^2+^-spike trains produced by CatSper and Ca_V_ channels in sea urchin sperm

**DOI:** 10.1371/journal.pcbi.1007605

**Published:** 2020-03-02

**Authors:** Daniel A. Priego-Espinosa, Alberto Darszon, Adán Guerrero, Ana Laura González-Cota, Takuya Nishigaki, Gustavo Martínez-Mekler, Jorge Carneiro

**Affiliations:** 1 Instituto de Ciencias Físicas, Universidad Nacional Autónoma de México, Cuernavaca, Morelos, México; 2 Instituto de Biotecnología, Universidad Nacional Autónoma de México, Cuernavaca, Morelos, México; 3 Instituto Gulbenkian de Ciência, Oeiras, Portugal; 4 Washington University School of Medicine, Department of Obstetrics and Gynecology, Center for Reproductive Health Sciences, St. Louis, Missouri, United States of America; 5 Centro de Ciencias de la Complejidad UNAM, CDMX, México; 6 Laboratoire de Physique Statistique, Départment de Physique, Ecole Normale Supérieure, Paris, France; Purdue University, UNITED STATES

## Abstract

Intracellular calcium ([Ca^2+^]_i_) is a basic and ubiquitous cellular signal controlling a wide variety of biological processes. A remarkable example is the steering of sea urchin spermatozoa towards the conspecific egg by a spatially and temporally orchestrated series of [Ca^2+^]_i_ spikes. Although this process has been an experimental paradigm for reproduction and sperm chemotaxis studies, the composition and regulation of the signalling network underlying the cytosolic calcium fluctuations are hitherto not fully understood. Here, we used a differential equations model of the signalling network to assess which set of channels can explain the characteristic envelope and temporal organisation of the [Ca^2+^]_i_-spike trains. The signalling network comprises an initial membrane hyperpolarisation produced by an Upstream module triggered by the egg-released chemoattractant peptide, via receptor activation, cGMP synthesis and decay. Followed by downstream modules leading to intraflagellar pH (pH_i_), voltage and [Ca^2+^]_i_ fluctuations. The Upstream module outputs were fitted to kinetic data on cGMP activity and early membrane potential changes measured in bulk cell populations. Two candidate modules featuring voltage-dependent Ca^2+^-channels link these outputs to the downstream dynamics and can independently explain the typical decaying envelope and the progressive spacing of the spikes. In the first module, [Ca^2+^]_i_-spike trains require the concerted action of a classical Ca_V_-like channel and a potassium channel, BK (Slo1), whereas the second module relies on pH_i_-dependent CatSper dynamics articulated with voltage-dependent neutral sodium-proton exchanger (NHE). We analysed the dynamics of these two modules alone and in mixed scenarios. We show that the [Ca^2+^]_i_ dynamics observed experimentally after sustained alkalinisation can be reproduced by a model featuring the CatSper and NHE module but not by those including the pH-independent Ca_V_ and BK module or proportionate mixed scenarios. We conclude in favour of the module containing CatSper and NHE and highlight experimentally testable predictions that would corroborate this conclusion.

## Author summary

Fertilisation in marine invertebrates, such as the sea urchin, occurs during broadcast spawning events in which males and females of co-localised species ejaculate sperm and spawn eggs synchronously. During these events, spermatozoa have to find and fertilise conspecific eggs in the midst of all the other ones, which is a remarkable navigation and mating choice achievement. Sperm cells do this by navigating towards the source of species-specific peptides released by the egg, steered by spatial and temporally orchestrated peaks in intracellular calcium concentration that trigger sudden reorientations. How these calcium spikes are regulated and timed remains elusive. Different calcium channels have been implicated by indirect experimental evidence giving rise to a complex network of putative interacting components. We gained insight into the structure and function of this network by modelling it as a set of candidate modules that could be studied separately. By using this ‘divide and conquer’ approach to the complexity of the network, we could characterise the potential dynamics of each module and confront these dynamics with specific quantitative data. Our results indicate that the channel mediating calcium signals in sea urchin sperm is likely CatSper, a calcium channel necessary for human male fertility.

## 1 Introduction

Intracellular calcium ([Ca^2+^]_i_) plays a key role as a second messenger in a wide variety of biological processes. Its elevation above resting levels conveys diverse physiological signals and may trigger cellular processes such as programmed cell death and egg activation. Both versatility and specificity of this ion as a physiological signal lie in how it is temporally and spatially organised within a cell [1]. Calcium oscillations are widely described in both excitable and non-excitable cells, and lie at the core of several signalling processes, such as neuronal firing, embryonic cell differentiation, immune cell activation and rhythmic beating of the heart. The mechanistic basis of these oscillations have been worked out in a number of cells. A good example is the calcium induced calcium release, which is generated by an inositol trisphosphate and calcium signalling system that controls a host of biological processes [2], for which its operation has been understood with the help of mathematical models [3, 4]. Furthermore, in olfactory neurons, calcium influx is regulated directly by cyclic nucleotides [5, 6].

The guidance of sea urchin spermatozoa towards their conspecific eggs during broadcast spawning events is orchestrated by [Ca^2+^]_i_-spike trains elicited by the chemoattractant sperm activating peptides (SAP) released by the eggs. These spike trains are decoded into a series of acute turns followed by straight swimming episodes that define the trajectory of the sperm cell. Preventing calcium influx from the external medium will abrogate the cell’s capacity to orientate in space. The sequence of spikes can be triggered by SAP in sperm that are fixed, showing a typical temporal organisation, characterised by a gradual decrease of the amplitude of the spikes and progressive increase of the interspike intervals.

The signalling network that mediates calcium-steered chemotaxis in sea urchin sperm has been an object of intense study, and several decades of experimental research have implicated multiple channels and molecular components [7, 8]. It is now clear that the first step leading to Ca^2+^ influx is an increase of intracellular cGMP concentration, which is synthesised in response to the natural ligand. Experiments in which the receptor activation was bypassed by releasing a caged analogue demonstrated that cGMP is sufficient to trigger the signalling network downstream [9]. The rise in cGMP leads to membrane hyperpolarisation by opening a cGMP-gated K^+^-channel (KCNG). How the hyperpolarisation is then transduced into Ca^2+^ flux dynamics is not fully established. It has been hypothesised [10, 11] that the membrane potential shift to more negative values would have two effects: to stimulate a depolarising cationic current by sperm specific hyperpolarisation-activated cyclic nucleotide-gated channels (spHCN) and to remove the inactivation of voltage-gated calcium channels (Ca_V_), which would then further depolarise the membrane. The depolarisation of the membrane would close spHCN and Ca_V_, allowing hyperpolarisation fluxes to predominate, repeating the cycle. This cycle would be the basis of the [Ca^2+^]_i_-spike train, the temporal organisation of which could be modulated by Ca^2+^-activated Cl^-^ and K^+^ channels. Another hypothesis [12] is that the cGMP-driven hyperpolarisation, by activating a Na^+^/H^+^ exchanger that extrudes protons, would rise the intraflagellar pH (pH_i_) eliciting Ca^2+^ influx by a pH-dependent Ca^2+^ channel, such as CatSper. This is a sperm specific calcium channel with unique features that separate it from the classical Ca_V_ family, including modulation of voltage-gating by pH_i_ and Ca^2+^. These features open the possibility for another negative feedback underlying [Ca^2+^]_i_ oscillations. The pH_i_ rise would promote the opening of CatSper that drives an upsurge in [Ca^2+^]_i_ and membrane depolarisation, which in consequence would inactivate respectively CatSper and the exchanger. The cycle would reinitiate once the membrane hyperpolarises again.

The evidence for the two types of calcium channels proposed to participate in SAP-activated [Ca^2+^]_i_-spike trains is summarised in Table 1. Most evidence is indirect and based on pharmacological approaches, which attempt to implicate channels using blocking and activating drugs that often are not specific and therefore have off-target effects. Genetic engineering techniques that would allow definitive conclusions on the necessity and sufficiency of the posited channels (e.g. null mutants) are not available in sea urchin. Therefore, it remains elusive whether a single or multiple types of calcium channels play an active role in sea urchin sperm signalling.

**Table 1:** Summary of experimental evidence on the main calcium channels proposed in the pathway activated by SAPs. ^a^It is worthwhile to mention that out of the pharmacological blockers listed, none of them are 100% specific to the associated channels. ^b^Antibody raised against a polypeptide of the *α* subunit in rat, which also recognises the homologous version in mouse and human sperm. ^c^Antibody raised against pore-forming subunit polypeptides of *A. punctulata* [12] and *S. purpuratus* [18]. ^d^ It is of note that direct electrophysiological recordings for this ion channel have not been achieved in mature sea urchin spermatozoa compared with the homologous complex in mouse, human and macaque spermatozoa [19].

Ca^2+^ channel	Experimental evidence on sea urchin sperm	
	Molecular biology	Pharmacology^a^
LVA (T type)	mRNA in testis tissue [10]	Nickel [13]
		Nimodipine [14, 15]
HVA (L type)	mRNA in testis tissue, immunolocalisation^b^ [16]	Nimodipine [16]
		Nifedipine [16]
		Verapamil [17]
CatSper^d^	mRNA in testis tissue, immunolocalisation^c^, mass spectrometry [12, 18]	Mibefradil [12]
		MDL12330A [12]
		NNC 55–0396 [18]

Mathematical modelling may help to explore alternative hypotheses that cannot be directly addressed and disentangled experimentally. The signalling network elicited by Speract in sea urchin sperm has been modelled using the discrete formalism [17, 20, 18]. While the initial discrete network models featured only Ca_V_s, a subsequent attempt to gain insights into the effect of multi-target niflumic acid suggested that pH-dependent CatSper would play a key role in Ca^2+^ oscillations, and that the calcium-dependent potassium channel (BK) would further modulate the spike trains [20]. The inherent limitations of discrete models in describing concentrations and kinetic details hinder the conclusions that can be drawn from comparison with quantitative experiments. A continuous model was used to identify the core components of the signalling network [21], implicating Ca_V_ channels, but the model was not directly compared to experimental data.

In the present work, we explore which putative calcium channels may mediate the Ca^2+^ response to SAP by describing the network using coupled ordinary differential equations that can fully harness the quantitative and kinetic information available in experimental data. We asked which channels may intervene, and how they may control the relevant dynamical features of the observed [Ca^2+^]_i_-spike trains. Our aim is to contrast the different hypotheses by their quantitative implications in order to identify critical properties that can be used to experimentally disentangle them.

## 2 Results

### 2.1 A modular signalling network

**Fig 1:**
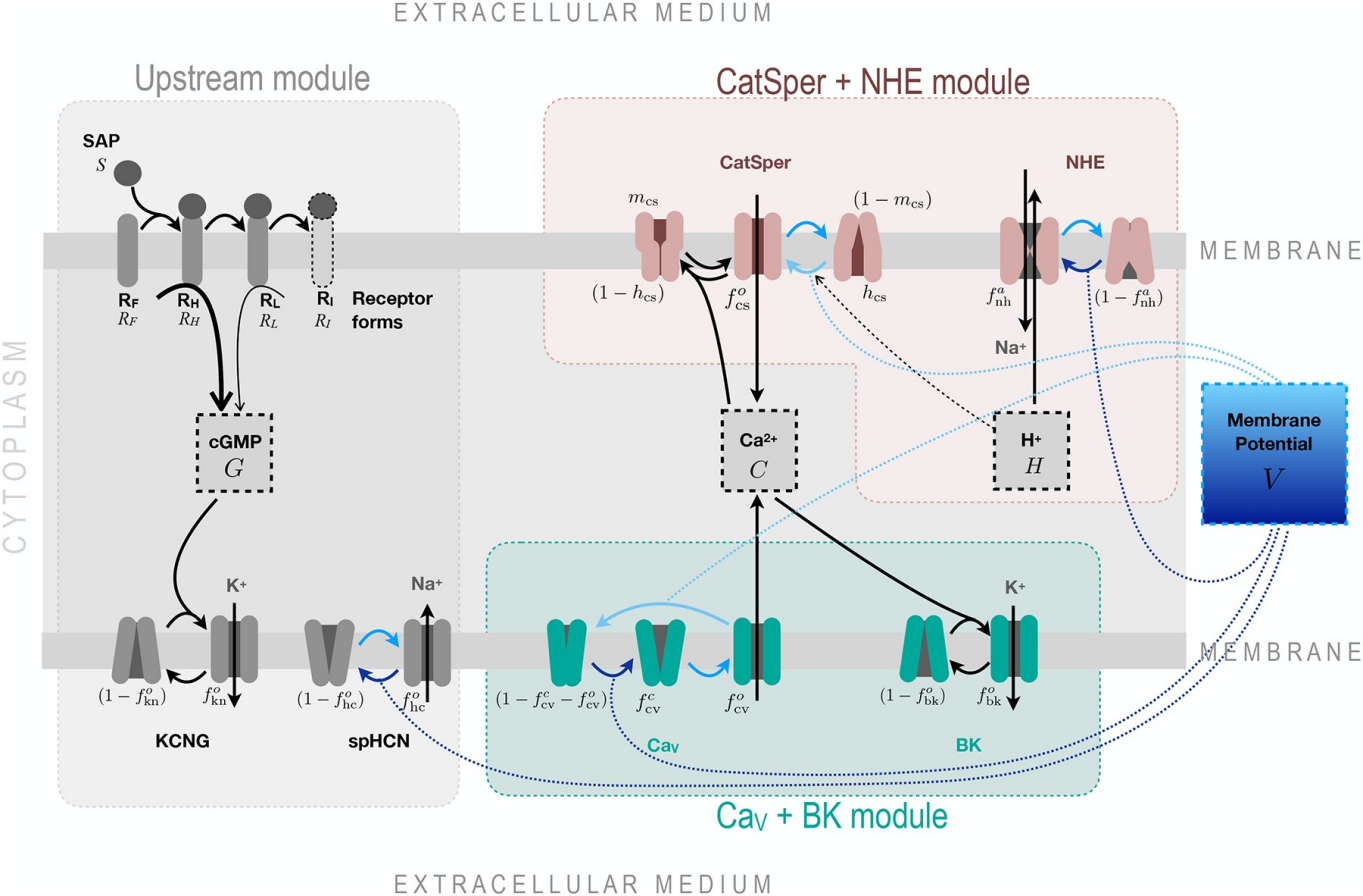
The modular organisation of the signalling network transducing SAP signals to Ca^2+^-spike trains. The structure is separated into 3 modules coupled by the membrane potential variable (*V*). In the Upstream module (described by the variables *S*, *R*_*F*_, *R*_*H*_, *R*_*L*_, *G*, $f_{\text{kn}}^{c}$, $f_{\text{kn}}^{o}$, $f_{\text{hc}}^{c}$ and $f_{\text{hc}}^{o}$) free receptors R_F_ bind SAP molecules and transit irreversibly through three receptor forms, each with less guanylate cyclase activity: High R_H_, low R_L_ and inactive R_I_; the cGMP synthesised by these receptors binds and opens KCNG channels, which conduct a hyperpolarising potassium current. The membrane hyperpolarisation promotes the opening of spHCN channels, which exert the opposite action on *V* when conducting a cationic inward current. Two candidate modules are presented to explain the calcium spike trains: one that includes classic voltage-dependent Ca^2+^ channels and BK channels (described by variables $f_{\text{cv}}^{c}$, $f_{\text{cv}}^{o}$, and $f_{\text{bk}}^{o}$), and another that considers CatSper, NHE exchanger and proton concentration (described by $f_{\text{cs}}^{o}=m_{\text{cs}}h_{\text{cs}}$, $f_{\text{nh}}^{a}$ and *H*). Note in the Ca_V_+BK module, that the calcium channel is purely voltage dependent, whereas CatSper in the other module has threefold regulation, namely Ca^2+^, H^+^ and *V*. The complexity of the CatSper channel is illustrated with two gates. Voltage-regulated channel transitions are depicted with blue arrows, where the darker or lighter tones indicate whether the transition is promoted by membrane hyperpolarisation or depolarisation respectively. The blue dotted lines linking some of these arrows to the membrane potential box top or bottom provide the same information. The components in the middle dashed boxes, namely cGMP (*G*), Ca^2+^ (*C*), proton (*H*) and membrane potential (*V*) are the main experimental observables. The ions K^+^ and Na^+^ are depicted in grey to indicate that the model describes only the currents while neglecting concentration changes.

The potential signalling network illustrated in Fig 1 is organised in three modules: an Upstream module, which includes the ligation of the receptor by SAP and the early transient drop of membrane potential *V* generated by the cGMP response, and two alternative downstream modules that can generate Ca^2+^ oscillatory responses. The Ca_V_+BK module includes voltage-dependent Ca^2+^-channels and Ca^2+^-dependent K^+^-channel BK; while the CatSper+NHE module is composed of the voltage-, pH_i_- and Ca^2+^-dependent channel CatSper, the voltage-dependent sodium-proton exchanger and the intraflagellar proton concentration. The variables, parameters and differential equations used to model the components of this network are described in Sec. 4.1 and Sec. 4.2.

The modular structure facilitated modelling and analysis. As a strategic simplification, we hypothesised that most of the components of the Upstream module were independent of the variables downstream and studied it isolated from the remaining dynamics. We tested the validity of this simplifying assumption by showing that this Upstream module accounted quantitatively for experimental time series of cGMP and membrane potential in response to SAP (Sec. 2.2). Once the values of the parameters of this module were obtained, they remained fixed in the subsequent analyses of the dynamics of the downstream components. In these analyses, the modules featuring Ca^2+^-channels were constrained such that given the input of the Upstream module, they generated an output that was consistent with the observed Ca^2+^-spike trains, i.e. these two putative modules were constrained by independent data sets at the both input and output levels. Hence, by coupling the downstream Ca_V_+BK and CatSper+NHE modules to this Upstream module, we show in Sec. 2.3 that either module can independently account for the observed Ca^2+^-spike trains patterns.

### 2.2 The Upstream module of the SAP-activated response explains the long term kinetics of cGMP independently of the downstream signals

**Fig 2:**
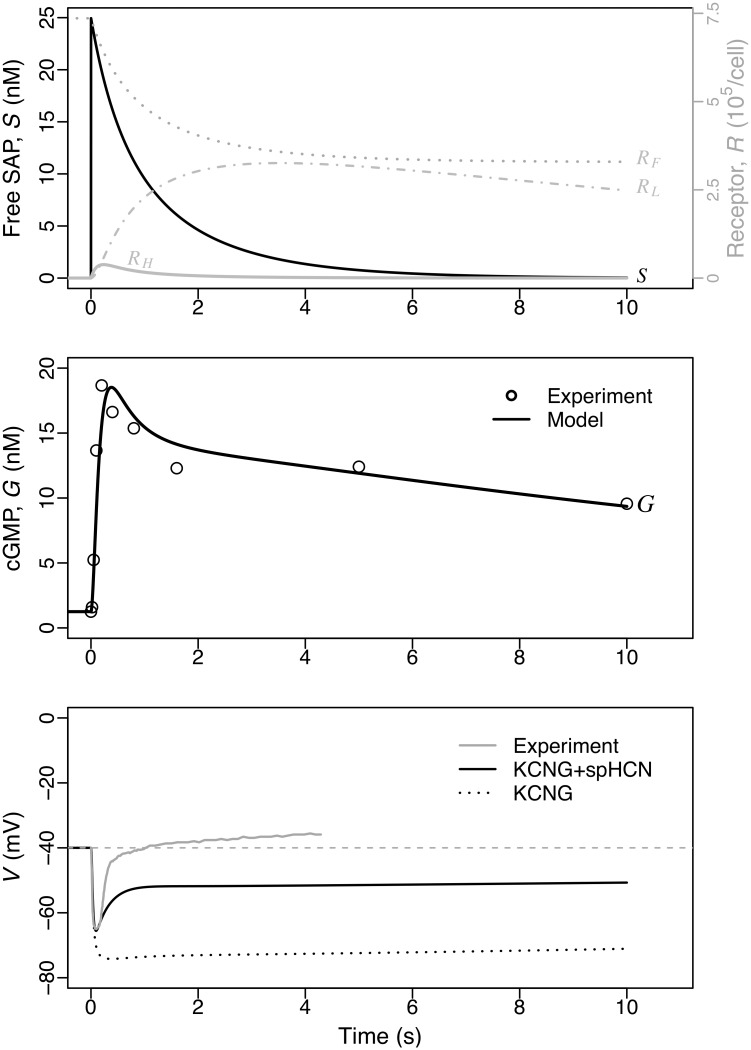
Numerical solution of the Upstream module overlaid with experimental data obtained in bulk sperm populations. The model with state space {*S*, *R*_*F*_, *R*_*H*_, *R*_*L*_, *G*, $f_{\text{kn}}^{o}$,$f_{\text{hc}}^{o}$, *V*}) was fitted to the cGMP concentration data measured on sperm populations stimulated at *t* = 0 with a SAP pulse *S*_0_ = 25 nM (data points read from the graphs in publications [22] and [10] for cGMP and *V*, respectively; see also S5 Fig). In the top graph, receptor activation dynamics and SAP consumption are shown. The middle graph displays cGMP concentration dynamics in the model (*G*, line) and the respective experimental measurements (dots) in nM. The bottom graph, shows two separate model solutions with KCNG+spHCN (solid black) and KCNG only (dotted; obtained by setting *g*_hc=0_). See main text for further details.

cGMP is the core component of the Upstream module (Fig 1). cGMP levels rise in response to SAP-induced guanylate cyclase activity and activates KCNG channels that in turn lead to hyperpolarising currents. *A. punctulata* sperm stimulated with the SAP Resact exhibit a rapid cGMP response that peaks before ∼400 ms and rapidly drops to a pseudo plateau that slowly decays over several seconds, as observed by stopped-flow kinetics [22]. This biphasic dynamics prompted us to postulate that SAP receptors transit irreversibly through three forms with different levels of associated guanylate cyclase activity, and that the guanylate cyclase and phosphodiesterases activities are insensitive to downstream signals (Fig 1; Sec. 4.2.1 and Sec. 4.1.2). We are thus neglecting putative regulation of phosphodiasterases activity by cGMP itself and by other components of the signalling network, such as pH_i_ or [Ca^2+^]_i_ [23]. In order to assess these assumptions we fitted a model of the Upstream module to cGMP measurements in bulk cell assays wherein sperm were stimulated with pulses of uncaged SAP along a concentration range spanning several orders of magnitude [22]. The good quality of the fitting (S1 Fig), involving appropriate scaling factors, supported quantitatively the assumptions and provided estimates of kinetic rate constants for receptor state transition, cGMP synthesis and decay (kinetics illustrated in Fig 2A for a SAP concentration that saturates the response of the membrane potential *V* [10]). The long term dynamics of the average cGMP level in the sperm population is described well by our single cell model, which as a first approximation neglected any potential feedbacks from downstream processes. This is our first modelling result.

By adjusting the density of KCNG and spHCN channels to membrane potential from sperm population data [10], we found values that reproduce the early transient drop in membrane potential and the early recovery (Fig 2). To illustrate the separate contribution of each channel, we showed a reference case where KCNG is the only channel present (dotted line in Fig 2) and, as expected, its opening alone drives membrane potential towards *E*_K_ ≈ −80 mV (potassium Nernst potential). The latter reference value sets a lower limit to the *V* response that is well below the value reached at the minimum *V* produced by the KCNG+spHCN case. It is worth noticing that under this scenario, in which only these two channels are assumed to be present, it is not possible to find a given combination of channel densities such that the spHCN current alone counterbalances the hyperpolarising current carried by KCNG, so as to bring membrane potential back to resting levels without abrogating the hyperpolarisation pulse. Therefore, full recovery of voltage requires the additional activity of other ion channels with depolarising currents after these initial events (namely the calcium channels).

### 2.3 Two alternative signalling modules can explain semiquantitatively the structure of the Ca^2+^-spike trains

**Fig 3:**
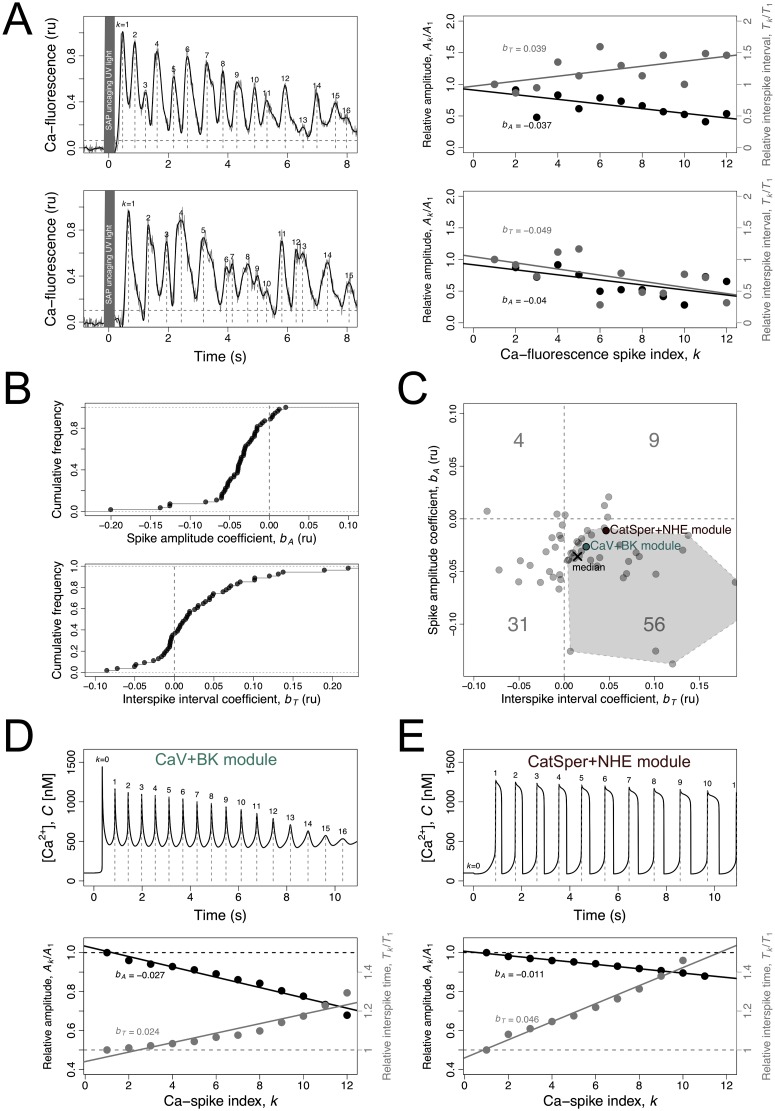
[Ca^2+^]_i_-spike trains produced by the sperm activating peptides in sea urchin sperm flagellum and its modelling. A) On the left, two examples are shown of [Ca^2+^]_i_ time series measured with fluorescent probes in flagella of *S. purpuratus* individual sperm bound to a coverslip following uncaging of Speract analogue by UV light. The raw measurements (grey) were smoothed using cubic spline (black). The Ca-spikes (vertical grey dashed lines) were calculated as the maxima in the smooth curve higher than 3x the standard deviation of the measurements pre-uncaging (horizontal gray dashed line). For each spike indexed *k*, the amplitude *A*_*k*_ and the interspike interval *T*_*k*_ = *t*_*k*+1_ − *t*_*k*_ were calculated. The shaded region indicates the UV light pulse. On the right, the relative spike amplitude *A*_*k*_/*A*_1_ (left axis; black) and relative interspike interval *T*_*k*_/*T*_1_ (right axis; grey) are plotted as a function the spike index *k* for the two examples. The lines are linear regressions of the relative spike amplitude (black) and relative interspike interval (grey) over the spike index *k* from which the respective regression coefficients *b*_*A*_ and *b*_*T*_ were obtained. B) Cumulative frequency distributions of the spike-amplitude regression coefficient *b*_*A*_ and interspike interval regression coefficient *b*_*T*_ for all experimental time series analysed. C) Bivariate plot of the spike amplitude coefficient *b*_*A*_ versus spike amplitude coefficient *b*_*A*_ in experimental time series (gray dots). The cross indicates the median values. The numbers indicate the percentages of time series falling in the four quadrants. The polygon in the bottom-right quadrant represents the convex hull of the experimental points in the quadrant where most the time series fall in. The pink and green dots represent the numerical solution of the model represented in D and E. D, E) [Ca^2+^]_i_ dynamics as predicted by the models featuring either the Ca_V_+BK module (green) (D) or the CatSper+NHE module (E), using the reference parameters (Table 2). On top, the curves represent the numerical solutions of the variable *C* as a function of time. The Ca-spikes are identified (vertical dashed lines) and indexed by *k* (labels). The relative spike amplitude and relative interspike interval are plotted as function of spike index *k* on the bottom graph together with the regression lines used to obtain the regression coefficients *b*_*A*_ and *b*_*T*_ for the model, which were represented in C.

The [Ca^2+^]_i_ spike trains observed in individual sperm cells responding to the SAP stimulus are noisy but show some conspicuous trends, as illustrated by those of *S. purpuratus* sperm (Fig 3A). The regression coefficients of the relative spike amplitude *b*_*A*_ and the relative interspike interval *b*_*T*_ over the spike index (illustrated on Fig 3A right) provide numeric measurements of these trends in the individual responses (Sec. 4.4). The amplitude of [Ca^2+^]-spikes declines progressively in 87% of the responses analysed (Fig 3B top), whereas the interspike interval tends to increase in 65% of the cases (Fig 3B bottom). The bivariate plot of *b*_*A*_ vs *b*_*T*_ (Fig 3C) shows that a majority of 56% of the [Ca^2+^]_i_ responses to SAP, as well as the median coefficients, fall within the bottom right quadrant. The convex hull defined by the experimental values that fall within this quadrant (the dashed polygon in Fig 3C) offers a first selection criterium to assess how close the dynamics predicted by the model are to the observations. The complementary criteria are the mean interspike interval and the steady state [Ca^2+^]_i_ value before the stimulus (Sec. 4.4). The rationale for the first selection criterium is twofold. First, it is the most representative pattern of the experimental spike trains analysed here as well as of those reported in other species (e.g. see fig. 3 in reference [9] for *A. punctulata* sperm). Second, it is the trend that was most difficult to obtain in the model under either the Ca_V_+BK or the CatSper+NHE modules, therefore being most suitable to challenge network composition. The spike trains falling in the bottom left quadrant, besides being less frequent experimentally, were easily obtained with the model and therefore were less challenging.

We found that the two alternative signalling modules, Ca_V_+BK and CatSper+NHE (Fig 1), can explain the characteristic envelope and increasing delay of the Ca^2+^-spikes when coupled alone to the Upstream module. These modules are composed of specific combinations of ion channels and transporters. Each of these combinations has the capacity to produce, given the input of the cGMP kinetics used to calibrate the Upstream module (Fig 2), a series of Ca^2+^-spikes that are coherent with the predominantly observed ones (Sec. 4.4). The corresponding numerical solutions (Fig 3D and 3E top) are characterised by amplitude and interspike interval regression coefficients (Fig 3D and 3E bottom) that fall within the values of the most likely observed responses in individual sperm cells of *S. purpuratus* (represented by the labelled circles in Fig 3C).

The two modules and their properties are analysed separately in the following sections. For each module, a first section characterises the Ca^2+^ oscillations and explains how they are generated (Sec. 2.4, Sec. 2.6), and a second focuses on explaining the progressive amplitude decay and delay between spikes (Sec. 2.5, Sec. 2.7). We will build on this knowledge to analyse and understand mixed scenarios (Sec. 2.8). The Ca_V_ channel was the first Ca^2+^ channel to be implicated in SAP signalling in sea urchin sperm literature and following this chronology we will start the analysis of its module.

### 2.4 Characteristics and mechanism of the [Ca^2+^]_i_ oscillations generated by the Ca_V_+BK module

The module features the Ca_V_ channels and the Ca^2+^-dependent BK potassium channels (Fig 1). The Ca_V_ channels transit irreversibly through three forms—inactive, closed and open—defining an ordered cycle, in which the transition rates are controlled by voltage. Only in the open form Ca_V_s allow inward Ca^2+^ currents that tend to depolarise the membrane. Provided that the transition rates between these forms are of similar order of magnitude, these channels alone give rise to a limit cycle, which as we will see has a constant period that is unrealistically short (Sec. 2.5). This disagreement with the observations called into action the other channel in the module, BK, which once coupled with the Ca_V_ results in larger progressively increasing intervals between the peaks and also increases the peak amplitude.

**Fig 4:**
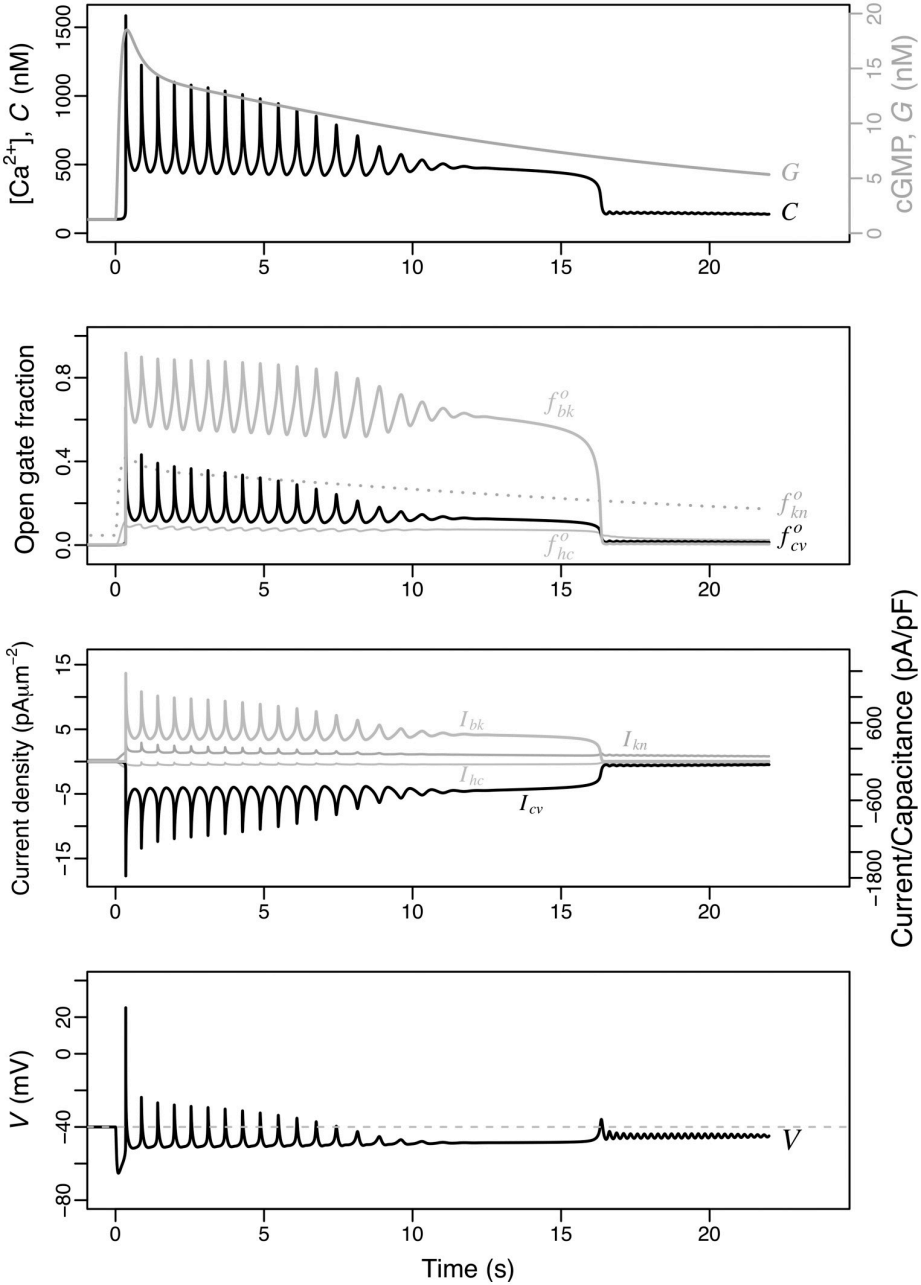
Dynamics of the model featuring the Ca_V_+BK module. The state space is {*S*, *R*_*F*_, *R*_*H*_, *R*_*L*_, *G*, $f_{\text{kn}}^{o}$, $f_{\text{hc}}^{o}$, *V*, $f_{\text{cv}}^{o}$, $f_{\text{cv}}^{c}$, $f_{\text{bk}}^{o}$, *C*}. As in Fig 2, the initial stimulus is *S*(0) = 25 nM is used. From top to bottom, the numerical solutions obtained with the parameters listed in Table 2 are shown for: [Ca^2+^]_i_ (*C*) together with cGMP concentration (*G*), open channel fractions, ionic currents and membrane potential. The ionic currents are displayed in current densities (left axis) and as current normalised by capacitance (right axis).

The numerical solution of the variables of the model including the Ca_V_+BK module under the reference parameters is shown in Fig 4. The uprise and slow decay of cGMP produced by the Upstream module leads to Ca^2+^-spike trains with an envelope that follows closely the cGMP values (top) and increasing interspike intervals. The initial transient hyperpolarisation (bottom) driven by the opening and currents of KCNG ($f_{\text{kn}}^{o}$ and *I*_kn_) precedes and triggers the first peak of [Ca^2+^]_i_ (*C*). The nadirs of the [Ca^2+^]_i_ oscillations are well above the basal concentration of this cation and this minimal plateau of about 500 nM is maintained until the Ca^2+^ activity vanishes at about 17 s. A similar oscillatory behaviour with similar period but different phases is observed on the fractions of open channels (second row) and the respective currents (third row), as well as the membrane potential (bottom).

The temporal dynamics of the [Ca^2+^]_i_ fluctuations predicted by this module can be easily understood qualitatively. The hyperpolarisation induced by KCNG enables the transition from inactive to closed forms of the Ca_V_ and the spHCN-mediated increase in membrane potential opens these closed channels. The open Ca_V_ channels will overcome the hyperpolarising current driven by KCNG and thus depolarise the membrane as Ca^2+^ ions flow in. The depolarisation of the membrane leads to the inactivation of the Ca_V_ channels with the hyperpolarisation currents predominating again. At the hyperpolarised membrane potentials the inactive channels will again transit to closed form, which eventually will open as described above, reinitiating the cycle. Intraflagellar calcium, by activating the BK channels, adds a strong hyperpolarising current to that of KCNG. This couples the net hyperpolarising currents to the amplitude of the previous Ca^2+^-spike, in such a way the higher the [Ca^2+^]_i_ peak, the higher the BK-driven hyperpolarising current will be.

### 2.5 Control of [Ca^2+^]_i_-spike amplitude and interspike interval in the Ca_V_+BK module

The envelope of the [Ca^2+^]_i_ spikes follows closely that of the cGMP levels upstream. This led us to speculate that the time course of cGMP might offer a way to understand the fluctuations envelope. To gain a better quantitative insight, a variant of the model was made wherein cGMP concentration, *G*, instead of a variable became a constant parameter, thus ignoring the upstream variables and reducing the state space (Sec. 4.3). In this simplified model, the qualitative changes of the [Ca^2 +^]_i_ and its period at the stationary states were analysed as a function of the *G* parameter. This is known as bifurcation analysis in dynamical systems theory, which is useful for characterising the global properties of a system of differential equations, in particular the dependence of asymptotic behaviours on a given parameter of interest (bifurcation parameter). These behaviours can correspond to attractors of fixed point (invariant in time) or limit cycles (stationary periodic), which in turn can be stable or unstable, among others. Wherever there is a qualitative alteration of these behaviours by changing the parameter of interest, it is said that a bifurcation has occurred.

**Fig 5:**
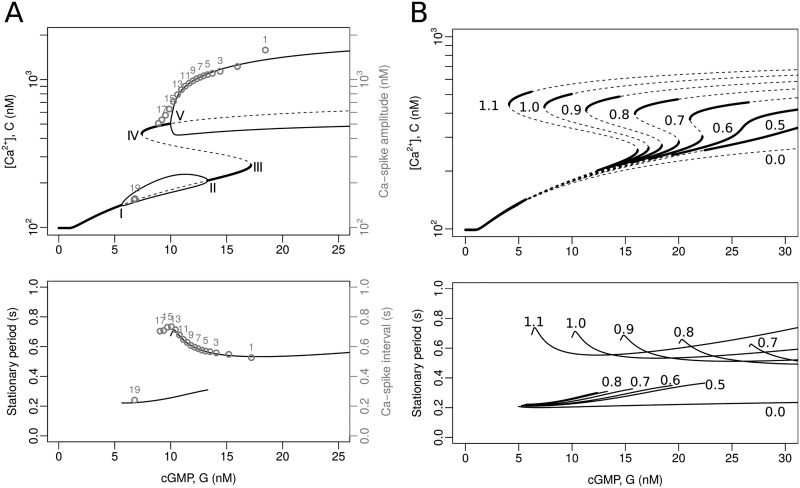
Bifurcation analysis of the model featuring the Ca_V_+BK module. In this analysis, cGMP concentration is a constant input, i.e. *G* is the bifurcation parameter and the upstream variables are ignored, thus reducing the state space to $\{f_{\text{kn}}^{o}$,$f_{hn}^{o}$, *V*, $f_{\text{cv}}^{o}$,$f_{\text{cv}}^{c}$, *f*_bk_, *C*}. A) Bifurcation diagrams calculated with XPPAUT [24] using the reference parameters (Table 2). Top: the graphs show the *C* variable ([Ca^2+^]_i_) as a function of the input parameter *G* (cGMP concentration). The thick continuous lines are stable equilibria, the dashed ones indicate unstable equilibria, the thin continuous lines are the maxima and minima of stable limit cycles. The bifurcation points are marked with Roman numerals (I to V). The numbered grey circles indicate the peak value for consecutive spikes obtained by numerically solving the complete model (see Fig 4). Bottom: the solid lines represent the period of stable limit cycles as a function of the constant input *G*. The numbered gray circles correspond to the interspike interval in the numerical solutions, as in the top graph. B) Bifurcation diagrams parameterised by maximal BK conductance density (*g*_bk_). Top and bottom: the lines are as in the graphs in (A); for clarity, the minima and maxima of the limit cycles are omitted. The numbers in the range of 0.0 to 1.1 are the fold change factors multiplying the reference value of *g*_bk_ (e.g. the curves labelled 1.0 coincide with those of (A), whereas the curves marked 0.0 correspond to a cell without BK channels).

When cGMP concentration is varied in this subsystem, the bifurcation diagram shows 5 bifurcation points (Fig 5A, labeled from I to V). In I, by means of a Hopf-type bifurcation, as cGMP concentration increases above the basal level, the system moves from a [Ca^2+^]_i_ stable stationary state to a limit cycle. When it reaches II, the limit cycle disappears giving rise again to a stable stationary equilibrium by means of an inverse Hopf-type bifurcation. Further on in III, the system undergoes an inverse saddle-node bifurcation, defined over cGMP concentration increments, in which a stable equilibrium state coalesces with an unstable one. In this way, in traversing the branch over the stable equilibrium state, the system goes to an unstable state. Now, when advancing on this last unstable equilibrium, this turns to a stable one by means of the direct saddle-node bifurcation (IV). The presence of points III and IV suggest the possible existence of a cusp bifurcation defined in a space of higher dimension, which is corroborated in panel B of the same figure. Finally, the last stable equilibrium state, again by means of a direct Hopf-type bifurcation, gives rise to another limit cycle.

The period of the limit cycle in the upper branch increases as the cGMP concentration decreases (Fig 5A, bottom), but this is not the case for the limit cycle in the lower branch, in which the period shows an opposite yet less pronounced trend. In addition, there is a considerable difference between the two limit cycles in terms of their amplitude, demarcated by the minima and maxima of calcium levels in the bifurcation diagram.

To better understand the role of the BK channel on calcium dynamics in a Ca_V_-dependent scenario, bifurcation diagrams were calculated within the cGMP concentration range considered in Fig 5A, for different density values of BK, represented by *g*_bk_ (Fig 5B). In B, unlike the upper panel of the Fig 5A, the lower and upper lines representing the limit cycles were omitted for clarity; their respective unstable steady state values are shown instead (dashed lines). In the bifurcation analysis, the extreme case where *g*_bk_ = 0, that is, the variant of the model that only presents Ca_V_, predicts the existence of a single branch for the calcium concentration with a limit cycle whose envelope is controlled by cGMP levels but has a constant period of approximately 0.2 s (see the lines labeled 0.0, in both panels of Fig 5). The gradual addition of BK reveals how bistability is generated (Fig 5B), and exhibits the appearance of an upper limit cycle within the range of cGMP concentration values observed in Fig 5A. The behaviour of the periods associated with the different values of *g*_bk_ is shown in the lower panel of the Fig 5B. In a three-dimensional representation, with *g*_bk_ as one of the axes, the bifurcation pattern is a cusp with mounted limit cycles. The presence of the new upper periodic attractor allows the nadir of the [Ca^2+^]_i_ fluctuations to be placed on a plateau above the basal level, and both their amplitude and increasing interspike interval to approach the physiological response. This implies that the aforementioned properties arise from the coupling of the Ca_V_ and BK channels.

The parameters have been chosen such that as the cGMP concentration values transiently exceed the bifurcation point, the system is forced to oscillate in the upper limit cycle and fall into the lower limit cycle after the decrease in cGMP causes the system to pass through the saddle-node bifurcation (IV). This explains the abrupt [Ca^2+^]_i_ drop and the low amplitude oscillations that persist afterwards, which are particularly visible in the numerical solution of *V* (Fig 4, bottom). If the value of *g*_bk_ were increased above the reference value used in panel A (e.g. by multiplying it by a factor of 1.1), the saddle-node bifurcation would occur at a lower cGMP concentration value and these low amplitude fluctuations would not be revealed (see Fig 5B).

Considering the bifurcations of the model obtained with cGMP as control constant, the behaviour of the full model is easy to understand: as SAP binds to its receptor, a sustained yet decreasing cGMP activity leads the system to approach asymptotically the attractors of the model with constant cGMP concentration, such that the system displays fluctuations whose spike amplitude and interspike interval tend to the amplitude and the period of the attractors (compare the numbered gray circles corresponding to the solution of the full system, which takes into account the temporal evolution of cGMP concentration as in Fig 4, to the asymptotic limit cycles for different fixed values in Fig 5A).

It is important to note that at intermediate levels of cGMP, there is a range of values where limit cycles coexist. From what has been reported in the literature, only the dynamics compatible with the upper limit cycle has been experimentally observed. Presumably, even if the small oscillations of the lower limit cycle were present, they would be masked by measurement errors.

### 2.6 Characteristics and mechanism of the [Ca^2+^]_i_ oscillations generated by the CatSper+NHE module

**Fig 6:**
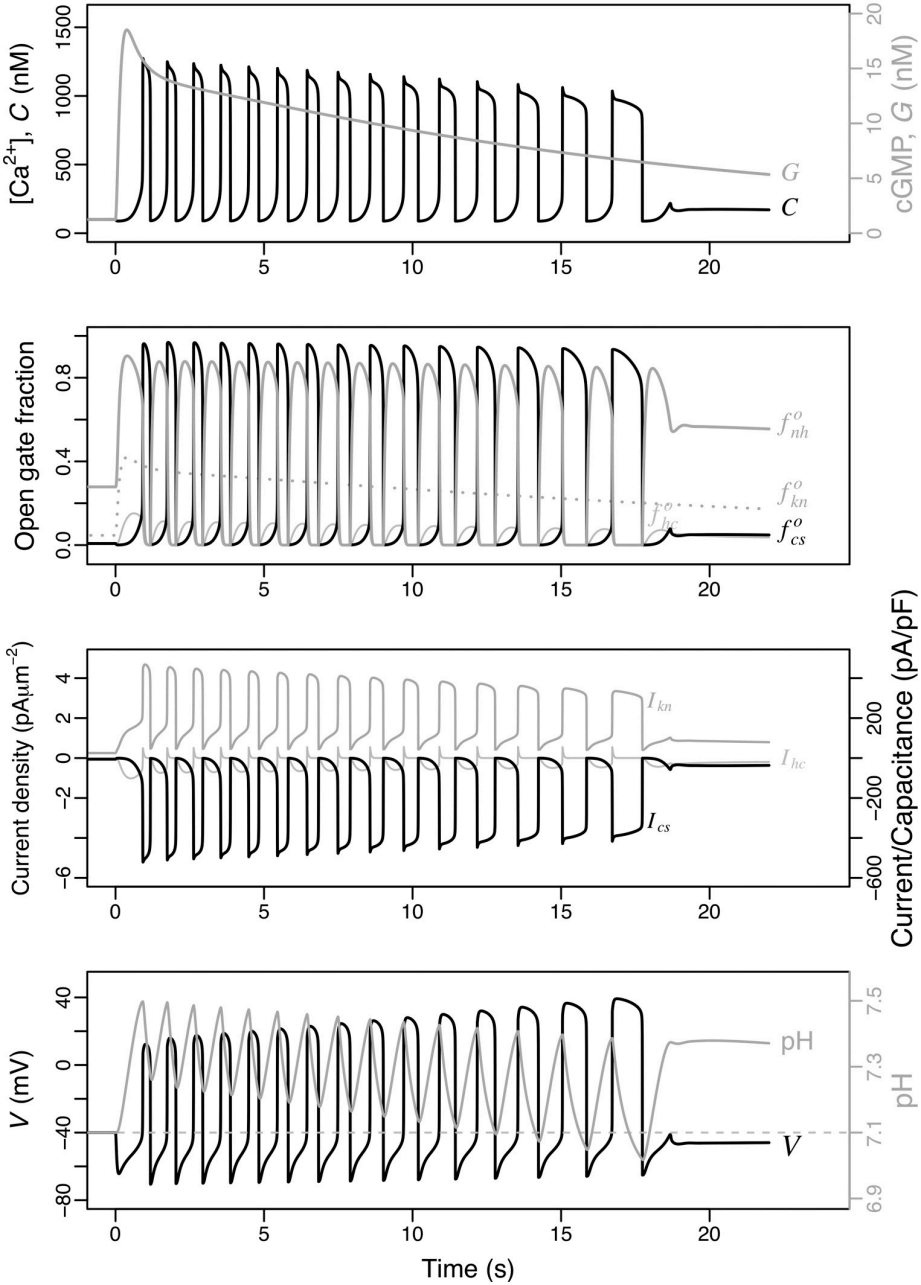
Dynamics of the model featuring the CatSper+NHE module. The state space is {*S*, *R*_*F*_, *R*_*H*_, *R*_*L*_, *G*,$f_{\text{kn}}^{o}$,$f_{\text{hc}}^{o}$, *V*, $f_{\text{cs}}^{o}$,$f_{\text{nh}}^{a}$, *C*, *H*}. As in Fig 4, the initial stimulus is *S*(0) = 25 nM, and the graphs are the numerical solutions for the indicated variables and intermediate quantities, obtained with the parameters listed in Table 2.

We now consider the alternative module whose central component is CatSper, the Ca^2+^ channel that has recently been implicated in SAP signalling in sea urchin sperm. We assumed here, that CatSper opening relies on two independent gates: one modulated by both pH_i_ and voltage, and the other one responsive to intracellular calcium. The fraction of open channels is given by the product of the fractions of these two open gates (Eq 44). The module includes also as variables the fraction of active forms of the voltage-sensitive Na^+^/H^+^ exchangers, $f_{\text{nh}}^{a}$, and the intraflagellar concentration of protons, *H*. The numerical solutions obtained under the same conditions as in the alternative model are shown in Fig 6.

The rise and fall of the cGMP produced by the Upstream module leads to a Ca^2+^-spike train with a slow decaying amplitude and an increasing interspike interval (Fig 6, top). The nadir of the Ca^2+^-oscillations is close or even below the resting level, in contrast with the oscillations predicted by the alternative model (Fig 4). A similar oscillatory behaviour with similar period but different phases is observed on the fractions of open channels (Fig 6, second row), the respective currents (third row), as well as the membrane potential and proton concentration (bottom).

Compared to the Ca_V_+BK module, the pattern of the Ca^2+^ oscillations is differently shaped by the CatSper+NHE module. The individual Ca^2+^-spikes are more prolonged and the corresponding nadir is relatively shorter. The decay of the spike train envelope is less pronounced for the reference parameters in the CatSper+NHE module than in the Ca_V_+BK module, while the increase in the interspike interval is more marked in the former than in the latter. It is remarkable that the CatSper+NHE module does not predict the sustained plateau of the Ca^2+^-oscillation nadirs present in the Ca_V_+BK module. Finally, another interesting difference between the two modules is the envelope of the voltage spikes whose amplitudes decrease progressively in the Ca_V_+BK module and increase in the CatSper+NHE module (bottom in Figs 4 and 6).

The basis for the limit cycle in CatSper+NHE module is qualitatively straightforward. The hyperpolarisation increases the activity of the Na^+^/H^+^ exchanger, this triggers the extrusion of protons raising pH_i_. As a consequence of this alkalinisation, the voltage sensitivity of CatSper shifts to lower values (see S3D Fig) and some of these channels open. The inward Ca^2+^ currents raise the membrane potential which in turn tends to increase the fraction of open channels, in a positive feedback loop. However, the progressive depolarisation of the membrane inhibits the exchanger and the pH_i_ tends to return to its basal level, shifting the CatSper voltage sensitivity back to higher values. This allows the hyperpolarising current to surpass the depolarising current of CatSper (see Fig 6, third row). The cycle restarts when the membrane becomes again hyperpolarised by the still ongoing KCNG current (Fig 6, second and third row), leading to a new round of enhanced exchanger activity, a transient rise in pH_i_, and the recovery of CatSper currents that eventually overcome the hyperpolarising currents. Although the feedback inhibition of CatSper by Ca^2+^-inactivation could have a potential role in the limit cycle, its contribution is actually negligible under the reference parameter regime.

### 2.7 Control of [Ca^2+^]_i_-spike amplitude and interspike interval in the CatSper+NHE module

**Fig 7:**
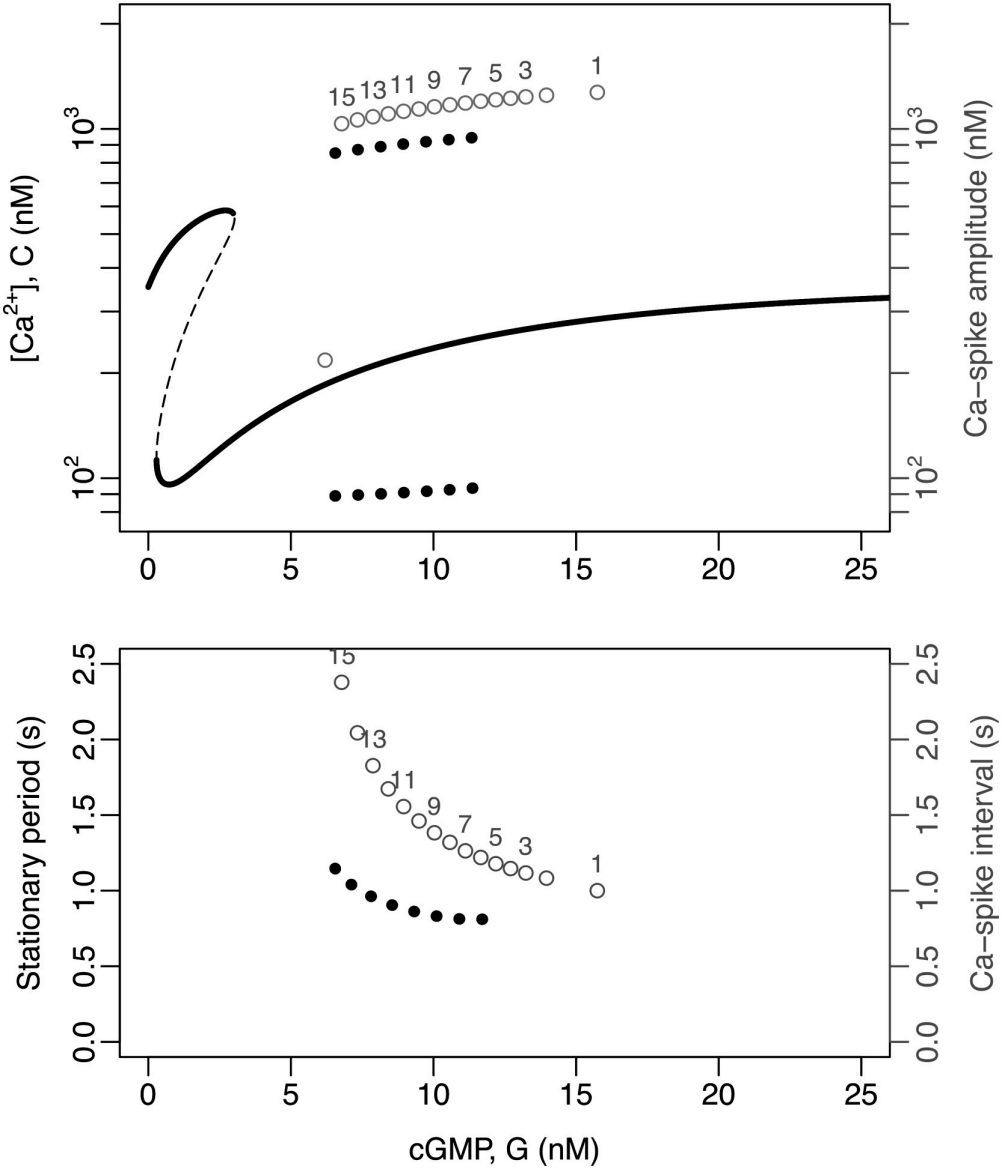
Bifurcation analysis of the model featuring the CatSper+NHE module. The analysis was done under conditions wherein cGMP is defined as a constant input (*G* = constant) and upstream variables ignored reducing the state space to $\{f_{\text{kn}}^{o}$,$f_{\text{hc}}^{o}$, *V*, $f_{\text{nh}}^{a}$, *m*_cs_, *h*_cs_, *C*, *H*}. Top: The graphs depict the variable *C* ([Ca^2+^]_i_) as a function of the input parameter *G* (cGMP concentration). The thick continuous lines are the stable equilibria and the dashed lines indicate unstable equilibria, as obtained by XPPAUT [24]. The thick dotted lines are the maxima and minima of the stable limit cycle obtained by numerical solutions of the system under random initial conditions. The numbered gray dots indicate the maxima and minima of the consecutive spikes obtained by solving numerically the full model, corresponding to those depicted in Fig 4. Bottom: The dotted lines represent the period of the stable limit cycles as a function of the constant input *G* obtained by numerical solutions. The numbered grey dots correspond to the interspike interval in the numerical solutions, as in the top graph.

As in the case of the Ca_V_+BK module, the bifurcation analysis of the model featuring the CatSper+NHE module with constant input cGMP reveals a rather complex dynamical structure. The system displays a cusp involving two saddle-node bifurcations, which, in contrast to the module Ca_V_ + BK, is in lower cGMP concentration ranges, such that two [Ca^2+^]_i_ stable equilibria coexist close to the resting state. At higher cGMP values, there is a regime where the stable equilibrium solution coexists with a stable limit cycle, whose maxima and minima are indicated by a succession of black circles in the top graph in Fig 7. It is worth noticing that the coexistence of stable attractors within a given range of cGMP concentrations suggests the presence of bifurcation points that determine the transition of one behaviour to the other, at the boundaries of that range. However, for this model, with the available bifurcation analysis software (e.g. XPPAUT [24]), it was not possible to discover these points or the limit cycle. In fact, the stable limit cycle was detected by solving the model numerically, exploring the phase space by randomisation of initial conditions of the system and allowing it to evolve for long times (>5 × 10^5^ iterations). Also an exploration of initial conditions between the two attractors, suggests that a possible presence of highly interlaced attraction basins, which makes numerical calculations difficult.

For this scenario, as was the case with the Ca_V_+BK module, the amplitude decreases and the period of the limit cycle increases (Fig 7, bottom) as the constant input of cGMP returns to basal levels after having reached high values.

The patterns of the [Ca^2+^]_i_ fluctuations obtained by solving the full model with the CatSper+NHE module (Fig 6) are now easy to understand. As the cGMP concentration increases rapidly from the resting state, the system falls within the basin of attraction of the limit cycle determined for constant *G* values; and as the cGMP returns slowly to its basal level, the time required to approach those limit cycles decreases. This can be seen in Fig 7 by means of the empty circles superimposed on the bifurcation diagram, which show how the system tends towards this limit cycle with a spike amplitude reduction and an increase in the interspike intervals. The differences between the asymptotic values of amplitude and periods predicted by this variant of the model with fixed values of *G* (black circles), and those of the actual peaks obtained when *G* varies in time (numbered empty circles) are more marked in this module than in the case of the Ca_V_+BK module.

### 2.8 The decaying envelope and temporal organisation of the Ca-spike trains can be obtained in mixed scenarios involving components of the Ca_V_+BK and CatSper+NHE modules

In the previous sections, we have shown that both alternative modules, once coupled with the Upstream module, can account for the observed data patterns. These two modules share some features and their components could potentially coexist in the flagellum being coupled through the membrane potential, pH_i_, and [Ca^2+^]_i_. As a first approach to investigate how the coupling of the modules would affect the dynamics, we considered a scenario in which the actual composition of the system would be a weighted combination of the two modules by means of weight parameters given by *θ*^cs^ = *θ* and *θ*^cs^ = (1 − *θ*) (implementation details in Sec. 4.5). For *θ* = 0, one recovers the submodel with CatSper module alone, for *θ* = 1 one recovers the submodel featuring Ca_V_+BK module only, and when *θ* = 0.5 the total conductance of the components in each module are half of their respective reference values. Taking the CatSper+NHE module we titrated in the components of the Ca_V_+BK module under several combination schemes. First, we explored the addition of Ca_V_ and BK simultaneously maintaining their conductance ratio as in the original Ca_V_+BK module (S4 Fig), then we added either channel alone (S5 Fig). This simple analysis showed that the modules cannot be combined freely without severely compromising the dynamics. Only in situations where one of the original modules largely predominates over the other (either *θ* ∼1 or *θ* ∼0) the mixed models predict the characteristic decay of the amplitude and the increase interspike interval. BK alone added to the CatSper+NHE module will cancel the oscillations at values as small as 10% of its reference value. At even lower densities it is functionally negligible, modulating the average interspike interval or the duration of the whole spike train without modifying the characteristics of the oscillations. These results are not entirely unexpected considering that in each oscillation of the Ca_V_+BK module the system spends most of the time hyperpolarised, while in the CatSper+NHE it spends a significant fraction of the cycle depolarised, which is an absolute prerequisite for pH_i_ to return to lower levels closing CatSper gate.

**Fig 8:**
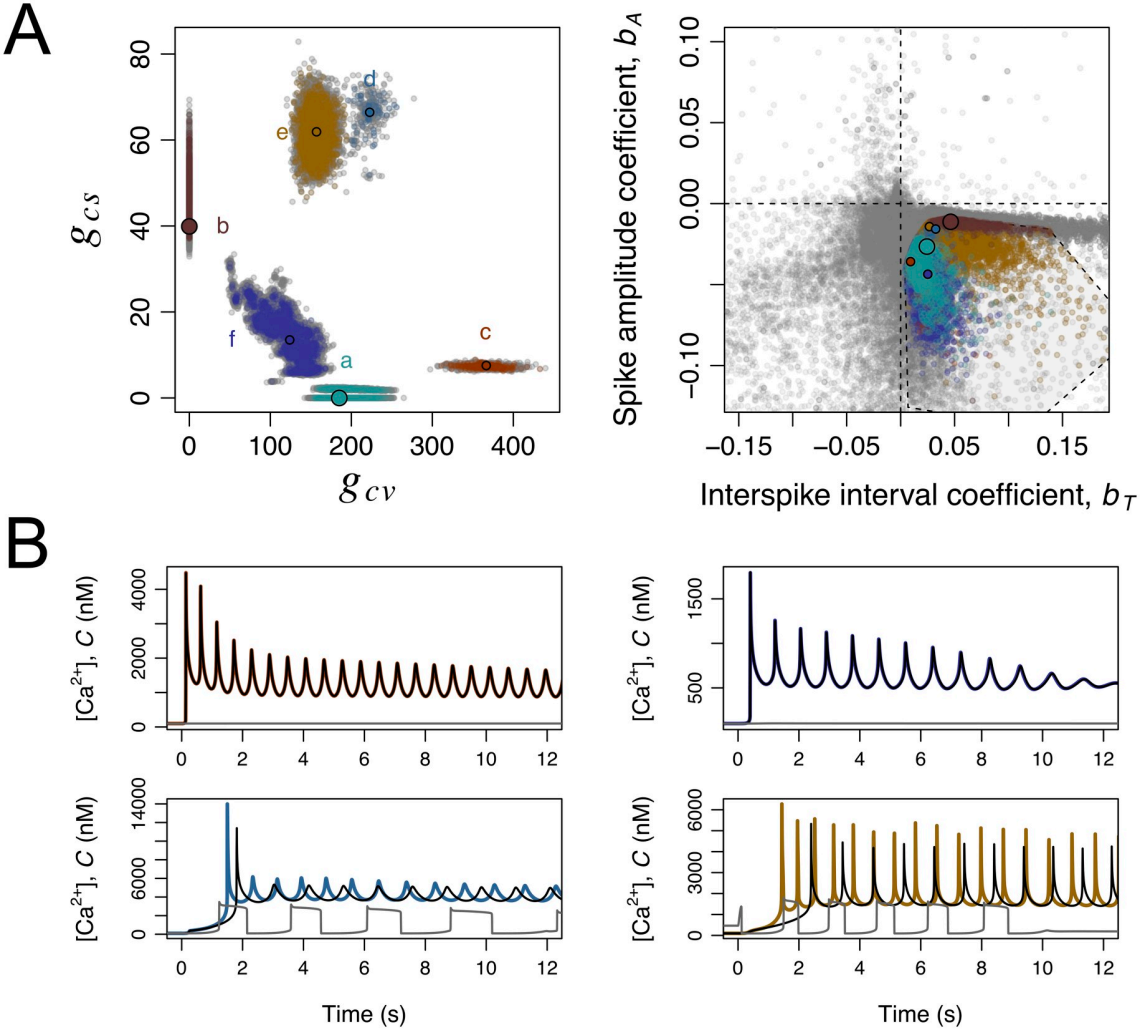
Exploration of mixed scenarios involving the components of the Ca_V_+BK and CatSper+NHE modules. The state space of the model was extended to {*S*, *R*_*F*_, *R*_*H*_, *R*_*L*_, *G*, $f_{\text{kn}}^{o}$,$f_{\text{hc}}^{o}$, *V*,$f_{\text{cv}}^{o}$,$f_{\text{cv}}^{c}$,$f_{\text{bk}}^{o}$,$f_{\text{cs}}^{o}$,$f_{\text{nh}}^{a}$, *C*, *H*}. A large number of parameter vectors resulting from a search of parameter space for coherence with experimental time series is illustrated in this figure according to clustering in the projection plane (*g*_*cv*_, *g*_*cs*_), predicted dynamics, and predicted functionality of the components of the two modules. During the exploration, coherence with experimental time series required that the resting *C*(0) is about 100nM, the average spike interval is in the range 0.37s-1.38 s, the predicted (*b*_*T*_, *b*_*A*_) point falls within the convex hull of most representative experimental values in the bottom right quadrant (polygon in A, right). A) On the left, the clustering of the parameter vectors in projection in the (*g*_cv_, *g*_cs_) plane is shown. On the right, the envelope and temporal organisation of Ca-spike trains predicted by each of these parameter vectors is represented as the bivariate plot of the spike-amplitude coefficient *b*_*A*_ vs the interspike interval coefficient *b*_*T*_. In both graphs the dots represent individual parameter vectors which are coloured according to the cluster if they are fully coherent with experimental time series (selected) and grey otherwise (unselected). For each cluster in the plane (*g*_cv_, *g*_cs_) a parameter vector has been singled out (smaller filled circles). The two larger filled circles represent the reference parameter vectors for Ca_V_+BK and CatSper+NHE modules. B) Illustration of the dynamics corresponding to the parameters vectors singled out in clusters c (top left), d (top right), e (bottom left) and f (bottom right) in A. The curves are the numerical solutions of the variable *C* in the model under the full parameter vector (coloured according to the cluster), setting *g*_cs_ = 0 (black) and setting *g*_cv_ = *g*_bk_ = 0 (grey). Notice the overlap between coloured and black curves on the graphs on top.

These considerations notwithstanding, the initial analysis of the mixed scenarios was done under a single parameter regime in all the remaining parameters, and it is possible that this may have constrained results. To investigate this possibility we explored the parameter space in search for mixed scenarios that would be coherent with the experimental Ca-spike trains based on the criteria defined in Sec. 4.4. The purpose was not to identify which parameters have the greatest impact on the models’ quantitative output, for which we could have resorted to Latin hypercube sampling and quantitative sensitivity analysis as done by others (e.g. [25, 26]). Our more immediate goal was to relax the parameter settings in order to detect potential mixed scenarios. Because our assessment of the model output is essentially a qualitative (binary) criterium of coherence with observations, the quantitative sensitivity analysis via partial rank correlation coefficients (as in [25]) or other methods (as in [26]) was not feasible. Based on these considerations, we devised a simple algorithm inspired on adaptive evolutionary dynamics to explore the parameter space. This algorithm (Sec. 4.5 and S1 Algorithm) evolved a seeding population of parameter vectors in a regular grid first neutrally and subsequently under stringent binary selection criteria. Variants of the parameter vectors were generated by a stochastic process, based on a random walk in logarithmic parameter space. The initial grid ensured that the abundances of the components of the Ca_V_+BK and CatSper+NHE modules were systematically explored in broad ranges, between negligible and excessive conductances. The generation of parameter vector variants ensured a random exploration of the parameter space centred on the realistic estimates of the other parameters, to avoid diversions into spurious parameter regimes. The final ensemble of parameter vectors produced by the algorithm contains both a population that was selected for being coherent with experimental data and an unselected population derived from and closely located to the former (Sec. 4.5 and S1 Algorithm). The projection of the parameter vectors in these two populations on the plane (*g*_*cv*_, *g*_*cs*_) shows 6 clusters, labelled a-f, depicted in Fig 8A. It is rather conspicuous that the cluster including the reference parameter set for the CatSper+NHE module (cluster b) is narrower than the other clusters. The cluster seems to be more sensitive to parameter changes as the within-cluster fraction of selected parameters is 8%. which is about half the average fraction of selected parameter vectors in the ensemble of the other clusters (16%). The mapping to the plane of the predicted spike amplitude and interspike interval regression coefficients indicates a narrow range of spike amplitude decay (Fig 8B). The other five clusters, including all mixed scenarios and the cluster centred on the reference Ca_V_+BK module, are more spread both in parameter space (*g*_*cv*_, *g*_*cs*_) and in the plane (*b*_*T*_, *b*_*A*_) summarising the predicted output. To understand the mixed scenarios we asked whether the presence of all the channels Ca_V_, BK or CatSper was essential to produce the predicted dynamics. To this end, we compared the numerical solution obtained with a given parameter set with the solutions obtained by specifically cancelling the conductance of Ca_V_ and BK on the one hand, or that of CatSper, on the other hand (examples of these numerical solutions are shown in Fig 8 for parameters singled out from each cluster). These comparisons revealed that CatSper has no functional consequences in the parameter vectors in clusters a, c and f whereas Ca_V_ and BK are irrelevant in those of cluster b. In clusters d and e, which include conductance densities of CatSper higher than the reference ones, the characteristic dynamics were severely distorted if either CatSper or Ca_V_ and BK were eliminated. In other words, the reference parameter set for the Ca_V_+BK module captures the essential qualitative features of the dynamics associated with clusters a, c and f and the reference CatSper+BK module represents well those of cluster b. However, the dynamics corresponding to clusters d and e may not be fully captured by the reference modules.

It is worth noticing that the numerical solutions obtained with the parameter vectors in the CatSper+NHE module cluster (cluster b) show in each oscillation cycle the typical preponderance of depolarisation, with blunt Ca-spikes and marked pH_i_ oscillations. The parameter vectors in the other clusters display very transient depolarisation times, with sharp Ca-spikes (Fig 8B) and pH_i_ oscillations with shallow amplitude around a relatively high value, qualitatively similar to those obtained with the reference Ca_V_+BK module (Fig 4). As we will show in the next section, this unique characteristic of the CatSper+NHE module cluster offers an avenue for model selection.

### 2.9 The CatSper+NHE module, but not the Ca_V_+BK module or any mixed scenario, predicts the cancelling of oscillations and sustained [Ca^2+^]_i_ following controlled alkalinisation

The two candidate modules for the signalling network activated by SAPs are, by construction, differently coupled to the pH_i_ dynamics. The NHE and [H^+^]_i_ can be coupled to the Ca_V_+BK module in the extended model by making *θ*^cv^ = 1 and *θ*^cs^ = 0 in Eqs 48–52. Under these conditions the exchanger is activated by hyperpolarisation and the proton concentration becomes an output variable, in the sense that pH_i_ does not feedback on any of the processes that determine Ca^2+^ fluctuations (proton concentration *H* does not appear in any of the equations that govern the variables of that model variant). Therefore, this module would predict that changes in the pH_i_ should not affect the Ca^2+^-spike trains. In contrast, in the CatSper+NHE module, the proton dynamics is tightly coupled to the Ca^2+^ oscillations and is an essential part of the mechanisms underlying the oscillatory behaviour. In our analysis of the parameter dependence of the CatSper+NHE module, we systematically found oscillations in [Ca^2+^]_i_ that were concomitant with pH_i_ oscillations having the same period, albeit phase differences. Parameters that lead to slower proton dynamics such that the pH_i_ oscillations vanish, resulted in cancellation of the oscillation in [Ca^2+^]_i_.

**Fig 9:**
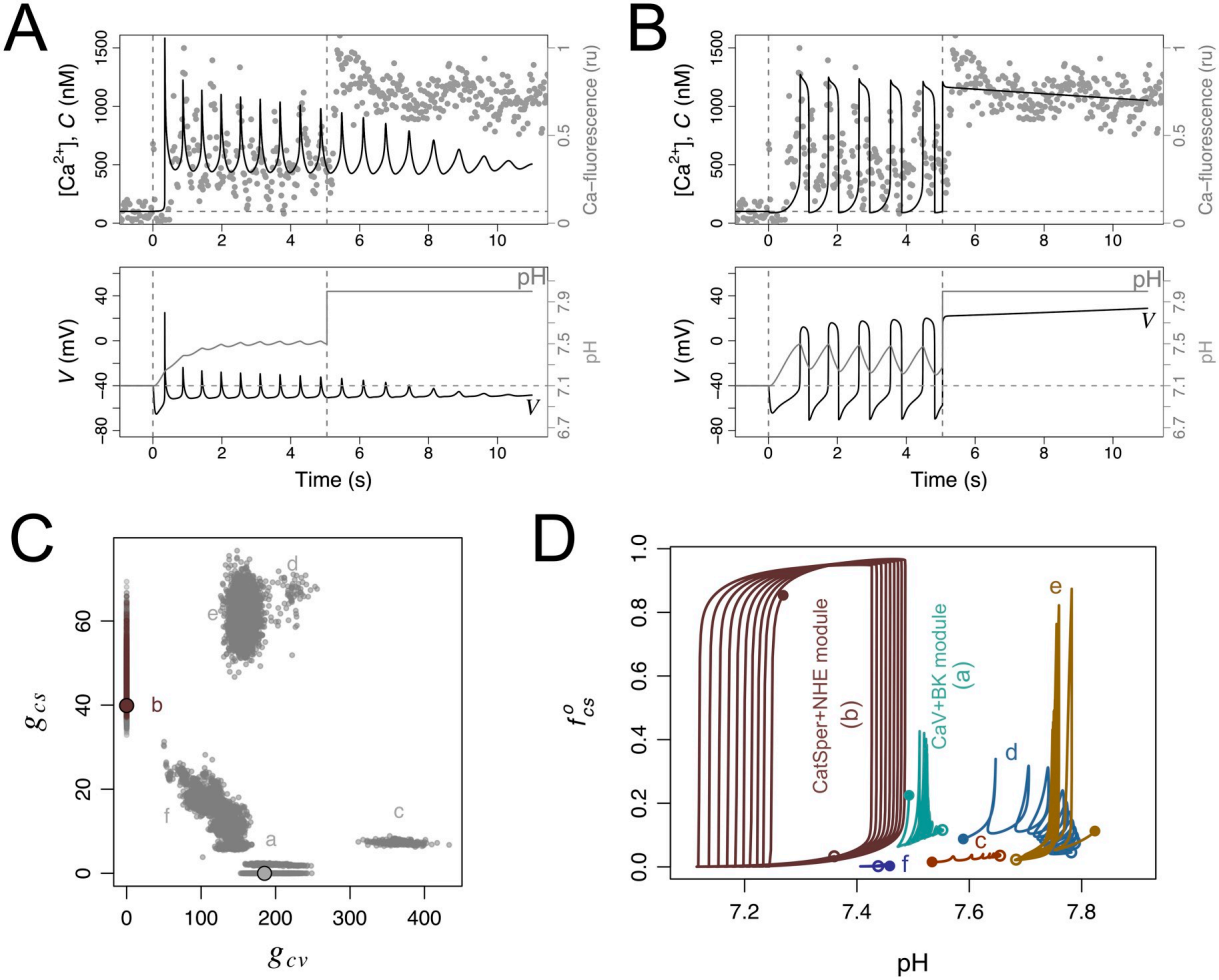
Responses to manipulation of pH_i_ in the reference Ca_V_+BK and CatSper+NHE modules, and in mixed scenarios. In A and B, the graphs show the numerical solutions of the indicated variables under the reference parameters of the Ca_V_+BK (as in Fig 4) and the CatSper+NHE (as in Fig 6) modules. In both cases, the normal response to SAP release at time 0 s (vertical dashed line) develops until it is perturbed by an artificial raise in pH_i_ (indicated by the second vertical dashed line) to a constant value maintained thereafter. Variables representing pH_i_ and NHE in the model were coupled to those of the Ca_V_+BK module with the same reference parameters used in the CatSper module. The grey dots are a rescaled trace of the relative intensity of a Ca-sensitive fluorescent probe in *S. purpuratus* individual sperm cell bound to a coverslip obtained by adding first 100 nM Speract (vertical dashed line) and subsequently 10 mM NH_4_Cl (second vertical line). C) Parameter vectors projected onto the plane (*g*_cv_, *g*_cs_) (as in Fig 8A) were coloured according to predicted model responsiveness during identical pH_i_ manipulation protocol: if alkalinisation results in sustained, slow decaying *C* dynamics the parameter set is representing according to the cluster’s colour, otherwise it is depicted in grey. D) Plot of the fraction of open CatSper channels $f_{\text{cs}}^{o}$ vs pH_i_ in the numerical solutions of the model parameterised under the reference CatSper+NHE module, the reference Ca_V_+BK module, and with the parameter vectors singled out from the clusters c, d, e and f illustrated in Fig 8. The trajectories are displayed for the interval 2-12 s to avoid transients.

The characteristics of the two modules imply distinctive responses to a manipulation that would artificially raise and fix the pH_i_ inside the flagellum. In this situation, the Ca_V_+BK module would predict that the Ca^2+^-fluctuations would be unaffected, while the CatSper+NHE module predicts that sustained pH_i_ increase would cancel [Ca^2+^]_i_ fluctuations and this cation would be sustained at higher concentration. These effects are illustrated in Fig 9A and 9B, where we also overlay the results of registering Ca^2+^ dynamics in the presence of ammonium chloride (NH_4_Cl), an agent that arguably raises the pH_i_ without affecting any of the other channels modelled here. As observed in the experimental trace, the exogenous alkalinisation gives rise to a dynamics that is qualitatively similar to the one predicted by the pH_i_-dependent CatSper+NHE module. The conspicuous effect of alkalinisation on [Ca^2+^]_i_ dynamics is not predicted by any of the mixed scenarios that were otherwise coherent with the experimental time series studied the previous Sec. 2.8 (Fig 9C). Although this result might appear surprising it has a simple rational. The oscillations in the CatSper+NHE module (as well as those obtained under all parameter vectors in cluster b) are characterised by and are strictly dependent on large amplitude alkalinisation and acidification cycles, which in turn require sufficiently long depolarisation in each cycle (Fig 9D). In contrast, the oscillations in the Ca_V_+BK module as well as those of the candidate mixed scenarios are characterised by very transient depolarisations resulting in sustained alkalinisation with shallow pH_i_ oscillations. In these cases, the concomitant oscillations in the fraction of open CatSper $f_{\text{cs}}^{o}$ are not driven by alkalinisation-acidification cycles and therefore manipulations that keep the cytosol in an alkaline state do not block the oscillations (Fig 9D).

### 2.10 Coexistence of two stable states in unstimulated sperm is predicted by the CatSper+NHE module and may be revealed by transient membrane depolarisation

**Fig 10:**
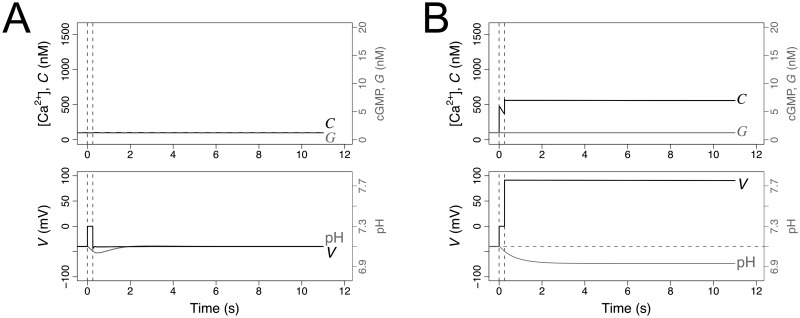
Ca_V_+BK (A) and CatSper+NHE (B) modules predict distinctive responses to manipulation of membrane potential. The graphs show the numerical solutions of the indicated variables under the reference parameters as in Figs 4 and 6. The normal resting state is perturbed (in the absence of SAP, *S* = 0 nM) by setting the membrane potential to a higher value (*V* = 0 mV) at time t = 0 for a short period of 0.25 s (indicated by the two vertical dashed lines).

The CatSper+NHE module is overall the most coherent with the experimental observations. Another of its most distinctive features is that it predicts the coexistence of two possible stable equilibria characterised by [Ca^2+^]_i_ at either basal levels or above basal levels. This latter state is further characterised for being more acidic and with higher membrane potential than the basal state. The coexistence of two states is best seen in the bifurcation diagrams in the top graph of Fig 7. According to the CatSper+NHE module, a sufficiently strong perturbation of the membrane potential may force a switch from the lower basal steady state to the state characterised by higher [Ca^2+^]_i_, where it will remain (Fig 10B). This behaviour contrasts with any model with a single resting stable state, such as the one with Ca_V_+BK module, in which the rise in membrane potential has, at best, transient effects on membrane potential or pH_i_ but not on [Ca^2+^]_i_, as illustrated in (Fig 10B). As a corollary of these properties, the Ca^2+^-spike train will always terminate in the basal level according to the Ca_V_+BK module, while according to the CatSper+NHE module, the [Ca^2+^]_i_ may remain at high values after the spike trains vanish under particular dynamics that favour depolarised membrane potential, as is the case of the time course depicted in Fig 6.

## 3 Discussion

This article explored the collection of channels that can explain the dynamics of the [Ca^2+^]_i_-spike trains elicited by SAP in sea urchin sperm. The large number of parameters (Table 2) used in the models studied here would deter any attempt of this kind, if it were not for the availability of quantitative time series of cGMP and membrane potential in response to several SAP concentrations. The parameters of the Upstream module that were not obtained from the literature were estimated by fitting the model to these time series data. This was possible given the structure of the signalling network in which the upstream components were assumed to be independent of the dynamics downstream and the fact that the model predicts a smooth non-oscillatory dynamics. The early signalling module was able to reproduce the long term temporal evolution of its output variable cGMP and also the early membrane potential changes in response to different SAP concentrations, measured in cell populations [10, 22]. This is remarkable considering that this module makes very simple assumptions about the receptor’s ligand binding and guanylate cyclase activity as well as about cGMP dynamics, neglecting adaptation mechanisms such as sensitivity readjustment of the receptor affinity that have been documented [27, 28, 29]. A noteworthy observation is that the cGMP dynamics at longer times, is characterised by a slow decay in which the cGMP concentration is in quasi-steady state following the slow decay of the receptor form with low guanylate cyclase activity (*R*_*L*_) (Sec. 4.2.1, Fig 2, first and second row). If the model predicted that cGMP itself would undergo periodic oscillations (arising potentially from feedbacks of pH_i_ on phosphodiesterase activity [30]), instead of a smooth dynamics, then we would lose the inferential power of fitting the predicted cGMP value to the mean population cGMP levels.

**Table 2:** Variables, intermediate functions, and reference parameters values.

Symbol	Description	Units	Reference value	Source
**Upstream module: SAP Receptor and cGMP signalling**				
SAP and receptor				
*S*	Extracellular SAP concentration	nM	Variable ^a^	Eqs 8–11 ^b^
*R*_*F*_	Number of free SAP receptors per cell (flagellum)	cell^−1^	Variable	Eqs 8–11
*R*_*H*_	Number of high activity SAP-receptor complexes per cell (flagellum)	cell^−1^	Variable	Eqs 8–11
*R*_*L*_	Number of low activity SAP-receptor complexes per cell (flagellum)	cell^−1^	Variable	Eqs 8–11
*R*_*T*_	Total number of SAP receptors per cell (flagellum)	cell^−1^	7.36 × 10^5^	F [22], S1 Fig
*θ*_*R*_	Receptor-bound to soluble SAP concentration conversion factor	nM cell	6.14 × 10^−5^	D (sec. 4.2.1)
*s*	Sperm density in measurement chamber	cell mL^−1^	3.706 × 10^7^	F [22], S1 Fig
*r*_1_	SAP-receptor association rate constant	nM^−1^ s^−1^	2.70 × 10^−2^	[50]
*r*_2_	*R*_*H*_ to *R*_*L*_ conversion rate constant	s^−1^	8.70	F [22], S1 Fig
*r*_3_	*R*_*L*_ inactivation rate constant	s^−1^	5.70 × 10^−2^	F [22], S1 Fig
cGMP				
*G*	Intraflagellar cGMP concentration	nM	Variable	Eq 4
*G*_*r*_	Resting cGMP concentration	nM	1.25	F [22]
*σ*_*G*_	Receptor-independent cGMP synthesis rate	nM s^−1^	23.65	F [22], S1 Fig
*δ*_*G*_	cGMP turnover rate constant	s^−1^	18.92	F [22], S1 Fig
*ρ*_*G*_	Receptor-dependent cGMP rate	cell^−1^ s^−1^	Function	Eq 14
*k*_*L*_	cGMP synthesis rate constant per *R*_*L*_	s^−1^	3.25	F [22], S1 Fig
*k*_*H*_	cGMP synthesis rate constant per *R*_*H*_	s^−1^	40.20	F [22], S1 Fig
*B*_*G*_	Flagellum cGMP buffering power	cell^−1^	5.49 × 10^3^	F [10]
*θ*_*G*_	Conversion factor to turn cGMP molecule number into effective intraflagellar concentration	nmcell	1.89 × 10^−4^	D (sec. 4.1.2)
KCNG channels				
$f_{\text{kn}}^{o}$	Fraction of open channels		Variable	Eq 15
*α*_kn_	Opening rate constant	nM^−1^ s^−1^	10	F [10]
*β*_kn_	Closing rate constant	s^−1^	257	[48]
*g*_kn_	Maximal conductance density	pS μm^−2^	135.30	A^c^ [10]
*I*_kn_	Current density	fA μm^−2^	Function	Eq 18
spHCN channels				
*m*_hc_	Fraction of open gates		Variable	Eq 19
$f_{\text{hc}}^{o}$	Fraction of open channels		Function	$m_{\text{hc}}^{3}$
${\hat{m}}_{\text{hc}}$	Steady state fraction of open channels		Function	Eq 20
*τ*_hc_	Characteristic time of gating	s	Function	Eq 21
*h*_1_	Half-activation voltage	mV	-50.80	[62, 76]
*h*_2_	Voltage sensitivity of activation	mV	6.60	[62, 76]
*h*_3_	*τ*_hc_ basal value	s	9.18 × 10^−2^	F [62], S2 Fig
*h*_4_	*τ*_hc_ amplitude	s	1.10	F [62], S2 Fig
*h*_5_	Voltage of maximum *τ*_hc_	mV	-42	F [62], S2 Fig
*h*_6_	Characteristic width of *τ*_hc_	mV	18.50	F [62], S2 Fig
*g*_hc_	Maximal conductance density	pS μm^−2^	193.50	A^d^ [10]
*I*_hc_	Current density	fA μm^−2^	Function	Eq 22
**Ca_V_ + BK module**				
Equilibrium parameters for *V* and *C*				
*g*_*L*_	Leakage conductance density	pS μm^−2^	1.94	F [10]^e^
*E*_*L*_	Reversal potential of leakage current	mV	80.05	D (Eq 3)
*σ*_*C*_	Ca_V_-independent Ca^2+^ source rate constant	nm s^−1^	1.14 × 10^8^	D (sec. 4.6)
*δ*_*C*_	Ca^2+^ extrusion rate constant	s^−1^	1.16 × 10^6^	MF
Ca_V_ channels				
$f_{\text{cv}}^{o}$	Fraction of open channels		Variable	Eq 30
$f_{\text{cv}}^{c}$	Fraction of closed channels		Variable	Eq 31
*α*_cv_	Inactivation recovery rate	s^−1^	Function	Eq 27
*β*_cv_	Opening rate	s^−1^	Function	Eq 28
*γ*_cv_	Inactivation rate	s^−1^	Function	Eq 29
*v*_1_	Inactivation recovery rate constant	mV^−1^ s^−1^	10.10	MF
*v*_2_	inactivation recovery rate voltage inflexion point	mV	-55	MF
*v*_3_	Inactivation recovery rate voltage sensitivity	mV	1.42	MF
*v*_4_	Opening rate constant	mV^−1^ s^−1^	33.80	MF
*v*_5_	Opening rate voltage inflexion point	mV	-39	MF
*v*_6_	Opening rate voltage sensitivity	mV	2.10	MF
*v*_7_	Inactivation rate constant	mV^−1^ s^−1^	8.10	MF
*v*_8_	Inactivation rate voltage inflexion point	mV	-18	MF
*v*_9_	Inactivation rate voltage sensitivity	mV	7.50	MF
*g*_cv_	Maximal conductance density	pS μm^−2^	185.16	MF^f^
*I*_cv_	Current density	fA μm^−2^	Function	Eq 33
BK channels				
$f_{\text{bk}}^{o}$	Fraction of open channels		Variable	Eq 45
*α*_bk_	Opening rate	s^−1^	Function	Eq 46
*β*_bk_	Closing rate constant	s^−1^	97	MF
*b*_1_	Opening rate constant	nM^−1^ s^−1^	0.97	MF
*b*_2_	Opening rate [Ca^2+^]_i_ inflexion point *α*_bk_	nM	316	MF
*b*_3_	Opening rate [Ca^2+^]_i_ sensitivity	nM	30	MF
*g*_bk_	Maximal conductance density	pS μm^−2^	214.50	MF^g^
*I*_bk_	Current density	fA μm^−2^	Function	Eq 47
**CatSper + NHE module**				
Equilibrium parameters for *V* and *C*				
*g*_*L*_	Leakage conductance density	pS μm^−2^	9	MF^h^
*E*_*L*_	Reversal potential of leakage current	mV	-20.4	D (Eq 3)
*σ*_*C*_	CatSper-independent Ca^2+^ source rate constant	nM s^−1^	3.72 × 10^7^	D (sec. 4.6)
*δ*_*C*_	Ca^2+^ extrusion rate constant	s^−1^	4.27 × 10^5^	MF
NHE exchangers				
$f_{\text{nh}}^{a}$	Fraction of active exchangers		Variable	Eq 23
*α*_nh_	Activation rate	s^−1^	Function	Eq 24
*β*_nh_	Inactivation rate	s^−1^	Function	Eq 25
*n*_1_	Activation rate constant	mV^−1^ s^−1^	1.51	MF
*n*_2_	activation rate voltage inflexion point	mV	-65	MF
*n*_3_	activation rate voltage sensitivity	mV	7	MF
*n*_4_	inactivation rate constant	mV^−1^ s^−1^	0.90	MF
*n*_5_	inactivation rate voltage inflexion point	mV	-30	MF
*n*_6_	inactivation rate voltage sensitivity	mV	7	MF
*J*_max_	Maximal H^+^ flux by total active exchangers	fmol s^−1^	3 × 10^−7^	MF
*K*_Na_	Na^+^ dissociation constant	mM	25	MF [69]^i^
CatSper channels				
$f_{\text{cs}}^{o}$	Fraction of open channels		Function	Eq 34
*m*_cs_	Fraction of channels with voltage- and pH-dependent gate open		Variable	Eq 35
*h*_cs_	Fraction of channels with Ca^2+^-dependent gate not inactivated		Variable	Eq 36
*v*_cs_	pH-dependent voltage leading to half-maximal gate opening at equilibrium	mV	Function	Eq 40
*s*_1_	Voltage-dependent gate opening rate constant	s^−1^	270	MF
*s*_2_	*m*_cs_ voltage sensitivity	mV	10	MF [12, 83], S3 Fig
*s*_3_	Amplitude of *v*_cs_ response to pH_i_	mV	79.70	F [72], S3 Fig
*s*_4_	pH_i_ leading to half-maximal *v*_cs_ value		7.20	MF [72], S3 Fig
*s*_5_	Hill-coefficient of *v*_cs_		35.52	F [72]
*s*_6_	pH_i_-independent, minimal *v*_cs_ value	mV	-41	MF, S3 Fig
*s*_7_	Scaling factor	nM	1	MF
*s*_8_	[Ca^2+^]_i_ leading to half-maximal *h*_cs_ inactivation (IC50)	nM	700	MF
*s*_9_	Hill coefficient of Ca^2+^-inactivation curve		2.50	MF
*s*_10_	Ca^2+^-inactivation rate constant	nM^−1^ s^−1^	1.40 × 10^−4^	MF
*g*_cs_	Maximal conductance density	pS μm^−2^	39.90	MF ^j^ [72]
*I*_cs_	Current density	fA μm^−2^	Function	Eq 44
**Common variables and parameters**				
*V*	Membrane potential	mV	Variable	Eq 1
*E*_*m*_	Resting membrane potential	mV	-40	[80, 84, 85, 10]
*C*_*m*_	Specific capacitance of flagellum membrane	pF μm^−2^	1 × 10^−2^	[86] ^k^
*v*_*f*_	Intraflagellar volume	fL	1.60	[48]
*s*_*f*_	Surface area of flagellar membrane	μm^2^	30	D ^l^
[K^+^]_o_	Extracellular potassium concentration	mM	9	[87] ^m^
[K^+^]_i_	Resting intraflagellar potassium concentration	mM	219	[66]
[Na^+^]_o_	Extracellular sodium concentration	mM	423	[87] ^m^
[Na^+^]_i_	Resting intraflagellar sodium concentration	mM	20	[81]
pH_o_	Extracellular pH		8	[87] ^m^
pH_r_	Resting intraflagellar pH		7.10	[81]
[Ca^2+^]_o_	Extracellular calcium concentration	mM	10	[87] ^m^
*C*_*r*_	Resting intraflagellar calcium concentration	nM	100	[81, 14]
*C*	Intraflagellar calcium concentration	nM	Variable	Eq 6
*H*	Intraflagellar proton concentration	M	Variable	Eq 5
*σ*_*H*_	NHE-independent proton source rate constant	M s^−1^	1.50 × 10^−7^	D (sec. 4.6)
*δ*_*H*_	Proton extrusion/consumption rate constant	s^−1^	1.28	D (Eq 5)
*E*_K_	K^+^ equilibrium potential	mV	-80	D^n^
*E*_hc_	Reversal potential of spHCN current	mV	-18	[61, 62, 63]
*E*_Ca_	Ca^2+^ equilibrium potential	mV	144	D^o^

^a^ The column entries are a numeric value if the quantity is a parameter or a label whether it is a variable or a function

^b^ The column entries: are a bibliographic reference unlabelled if the values were used as reported in the original publication or with labels F and MF if further processed; a derivation labelled D followed by a reference to the section where its derivation is explained; or a reference to the defining equation(s) defining a variable or function.

^c^ Taking into account the membrane surface of the flagellum *s*_*f*_, and KCNG unitary conductance (110 pS [59]), this implies ∼37 channels per flagellum

^d^ Taking into account the membrane surface of the flagellum *s*_*f*_, and spHCN unitary conductance (43 pS [63]), this would imply ∼135 channels per flagellum

^e^ Fitted in the Upstream module

^f^ Taking into account the membrane surface of the flagellum *s*_*f*_, and Ca_V_ unitary conductance (4.70 pS [82]), this implies ∼1182 channels per flagellum

^g^ Taking into account the membrane surface of the flagellum *s*_*f*_, and BK unitary conductance (70 pS [42]), this implies ∼92 channels per flagellum

^h^ Fitted in the complete CatSper + NHE module to conserve the initial drop of membrane potential, whereas allowing calcium signal to return to levels close to resting value after spike-train finishes

^i^ Based on figure 1 of [69]

^j^ Taking into account the membrane surface of the flagellum *s*_*f*_, and assuming that the Ca^2+^ unitary conductance of CatSper might be below 1 pS, this implies ∼1200 channels per flagellum

^k^ Conventional value for eukaryotic cytoplasmic membranes

^l^ Calculated as the lateral surface of a cylinder with length 40 μm and diameter 0.240 μm

^m^ Typical concentration in artificial seawater used for embryology experiments on marine organisms

^n^ Calculated with the Nernst equation, $E=\frac{RT}{zF}ln\frac{{\left[X\right]}_{o}}{{\left[X\right]}_{i}}$, having temperature *T* = 17°C

^o^ Calculated with the Nernst equation, setting temperature T = 17°C, and using the *C*_*r*_ parameter as internal concentration value

The Upstream module alone was able to explain the early hyperpolarisation dynamics based on the joint action of KCNG and spHCN channels (Fig 2, bottom). The parameters were set such that, in the absence of any other channels, the repolarisation initiated by spHCN following the hyperpolarising current of KCNG, leads to a partial recovery of the membrane potential that remains below the resting value. The partial recovery implies that spHCN effective conductance density is sufficient to set into motion the downstream components (e.g. the opening of closed Ca_V_ channels or shifting to higher opening rate of the voltage-gate of CatSper) but not too strong to prevent the oscillatory [Ca^2+^]_i_ dynamics. This property of the signalling network is amenable to experimental corroboration by assessing whether the membrane potential recovery is only partial under a setting in which all calcium entry is prevented by using zero external concentrations of this cation.

The upstream SAP-induced cGMP response curve is linked to the downstream [Ca^2+^]_i_ fluctuations by the dynamics of the network. The key question was: what components of the downstream modules correctly predict this link? Our analysis indicates that either one of two modules featuring voltage-dependent channels could be responsible for the dynamics of [Ca^2+^]_i_ fluctuations. The first module calls into action Ca_V_ channels that undergo voltage-dependent sequential transitions from inactivated to closed, to open and back to inactivated states. These irreversible transitions bring forth a limit cycle with constant period with an amplitude that is controlled by cGMP activity. These Ca_V_ channels, which are predominantly inactivated at the resting potential, are released from inactivation upon a transient hyperpolarisation and subsequently opened when spHCN channels activity raises the membrane potential. In this module, the oscillations are strongly modulated in amplitude and tempo by the Ca^2+^-dependent channel BK to produce the experimentally observed progressive increase in the intervals between [Ca^2+^]_i_ spikes. The oscillations predicted by this module vanish when cGMP falls below a critical value involving a Hopf-type bifurcation (Fig 5A).

The second module capable of explaining the organisation of the [Ca^2+^]_i_ fluctuations features a single channel, CatSper, that is voltage- and pH-dependent and is reversibly inactivated by intracellular Ca^2+^ ions. In contrast with the previous module, which is set in motion directly by the effect of transient hyperpolarisation that relieves Ca_V_ channels from inactivation, the initial opening of CatSper channels is triggered indirectly by alkalinisation of the cytoplasm driven by the hyperpolarisation-dependent Na^+^/H^+^-exchanger activity (S3 Fig). The cycling behaviour is produced by the negative feedback that depolarisation driven by CatSper activity has on inactivating the Na^+^/H^+^-exchanger, reducing pH_i_ and decreasing CatSper activity. Another feedback arising from the inactivation of CatSper by [Ca^2+^]_i_ plays a more preponderant role in controlling the length of the Ca^2+^-spike train and in increasing the interspike intervals. As in the case of the first module, the oscillations generated by the module stop when the cGMP activity falls below a critical value (Fig 7).

It is noteworthy that, under the reference parameters used in this article, the overall temporal scale of the calcium response produced by the CatSper+NHE module is slower compared to the experimental tracings, in terms of real time units; however, if the relative progressive increase of interspike intervals is contrasted instead, this property becomes more marked in this model than in the one featuring the Ca_V_ + BK module, and more closely related to the experimentally observed (Fig 3C). In the numerical solutions with the former module the interval between the last spikes can be two or three fold larger than the interval between the first spikes in the train, as observed in the experiments in single cells. A comparable increase in interspike intervals can be obtained with the Ca_V_+BK module under different parameter vectors (such as those in cluster a). Conversely, the progressive decrease in the amplitude observed experimentally is better accounted for by the Ca_V_+BK module than by the CatSper+NHE module under all the parameters studied here.

These two downstream modules confer some distinguishable properties to the signalling network when coupled to the Upstream module. One of those properties is the envelope of the membrane potential oscillations. In the module featuring the Ca_V_ + BK channels pair. This envelope has a shape similar to that of the [Ca^2+^]_i_ spikes. In contrast, the CatSper+NHE module predicts that the amplitude in the membrane potential peaks should increase until it suddenly ceases (Fig 6, bottom), thus establishing an opposite trend to the calcium-spike train envelope. Interestingly, the ascending *V* envelope is more similar to the observed at prolonged times in membrane potential measurements of sperm populations stimulated with high doses of SAP [10].

Another good example of distinctive features between the alternative scenarios is the permissive cGMP range that allows for oscillatory solutions. At too low (close to resting) or too high intracellular GMP concentrations, the model with CatSper predicts a sustained rise [Ca^2+^]_i_ without noticeable oscillations (Fig 7). Conversely, in the Ca_V_ + BK module, the periodic regime is maintained for any value above a critical level of cGMP, however, above more elevated concentrations, the amplitude and period saturate to nearly constant values (Fig 5A). These properties of the model could be addressed experimentally by phosphodiesterase inhibitors or by elevating cGMP to saturating concentrations by uncaging an analogue. It is worth recalling the reports that intracellular uncaging of cGMP leads to an increase in [Ca^2+^]_i_ that is oscillatory in the case of *A. punctulata* [9, 31] but transient in the case of *S. purpuratus* ([15]) spermatozoa. The discrepancy between the two species could be reconciled if there are species-specific differences in the boundaries of cGMP intervals that produce oscillations or alternatively by experimental differences in the uncaged concentration of the cyclic nucleotide.

The difference between the two alternative network modules that is most decisive is the distinct interplay with cytosolic pH and proton dynamics. While the periodic alkalinisation and acidification is an essential part of the limit cycle characteristic of the CatSper+NHE module network, proton dynamics is secondary and inconsequential in all the networks that included the alternative Ca_V_ + BK module. For this reason, only the CatSper+NHE module predicted that externally forced cytosolic alkalinisation should lead to sustained [Ca^2+^]_i_ as observed experimentally following the addition of NH_4_Cl to *S. purpuratus* spermatozoa (Fig 9A). This decisive results prompts us to conclude that the channel responsible for the [Ca^2+^]_i_ responses to SAP is the pH-dependent CatSper instead of Ca_V_. However, this conclusion, does not follow simply from the pH sensitivity of CatSper or from the SAP induced alkalinisation. These features were also present in the mixed scenarios that failed to reproduce the response to forced alkalinisation, despite being compatible with the other observations.

Further considerations on this conclusion are called for. One of its corollaries is that observing [Ca^2+^]_i_ oscillations dependent on CatSper and NHE implies necessarily the concomitant observations on pH_i_ oscillations with the same period. Oscillations of pH_i_ with the same period of those of [Ca^2+^]_i_ were not detected in experiments where the levels of pH_i_ have been monitored in individual cells [32]. This might reflect limitations of the experimental system (e.g. too low signal to noise ratio of the pH probe as compared to the Ca^2+^ probe, particularly if the relevant proton concentration is the one near the membrane and not in the bulk of the flagellum) or, alternatively, it might mean that intraflagellar pH is not oscillating periodically. In the latter case, the hypothesis that CatSper is the main responsible for fluctuating Ca^2+^ currents, at least in the way we modelled it here, is questionable and must be reexamined opening a new avenue for modelling research. In this context, it might be relevant to note that oscillatory fluxes of Ca^2+^ and protons have been reported with the same period as growth oscillations of pollen tubes (reviewed in [33]).

The observation that a rise in pH_i_ precedes the first Ca^2+^ spike in sea urchin sperm stimulated by SAP has been interpreted as an indication that Ca^2+^ currents are a consequence of this early alkalinisation [34, 12, 32]. This interpretation became a cornerstone of the hypothesis that pH-sensitive CatSper channel is responsible for Ca^2+^ fluctuations. It is worth calling attention to the early time courses of pH_i_ and [Ca^2+^]_i_ obtained by the numerical solution of the model featuring the Ca_V_+BK module (Fig 9A). In this model, where Ca^2+^ currents are, by construction, independent and unaffected by pH_i_ one can nevertheless observe an onset of alkalinisation that precedes the first [Ca^2+^]_i_ spike. This illustrates the frailty of inferring causation from time series data in general and emphasises the need to support the hypothesis that a pH-sensitive Ca^2+^ channel is involved responses to SAP with more decisive experiments such as the controlled pH_i_ manipulation, as proposed here.

Another key prediction of the CatSper+NHE module is the existence of bistability at the resting values of cGMP. This implies that perturbations exist that may lead to a sustained [Ca^2+^]_i_ value above the normal resting one, in the complete absence of SAP signals. We illustrated this by a transient rise in membrane potential that locked the system in the stable state characterised by higher, tonic-like [Ca^2+^]_i_ (Fig 10B top). Another perturbation with comparable effects would be a transient yet complete depletion of cGMP that would push the system across a bifurcation point in which the lower equilibrium vanishes. Following such perturbation, hysteresis is predicted such that the system could remain in the higher [Ca^2+^]_i_ state after restoration of the basal cGMP levels. The CatSper+NHE module bistability also implies that after a [Ca^2+^]_i_-spike train terminates, the system may or may not return to the basal level. It is interesting to note that this alternative [Ca^2+^]_i_ equilibrium state also entails low pH_i_ levels and depolarised membrane potential (Fig 10B bottom). If such a state existed, its physiological meaning would be associated with a state of quiescence, since the acidification of the cytoplasm below the basal pH_i_ inhibits both the metabolism and the dyneins, which are the molecular motors that drive the flagellar beating [35, 36]. On the other hand, the viability of sperm would be affected in this state, since sustained high levels of calcium represent a universal signal that triggers cell death in the majority of eukaryotic cells [37, 38, 39]. These properties of bistability and hysteresis at cGMP levels close to the resting state are not present in the Ca_V_+BK module, thus offering yet another way of teasing apart the two hypotheses.

Our analysis indicates that proportionate mixed scenarios involving components of the two modules are implausible in the sea urchin sperm. The Ca_V_ and BK channels can fine-tune the temporal structure of spike trains elicited by CatSper if present at minute densities. At densities or currents comparable to those of CatSper these channels will disrupt the [Ca^2+^]_i_ oscillation train or bring forth an oscillatory regime in which the proton dynamics plays an irrelevant subsidiary role. In the same vein, the CatSper+NHE module predicts [Ca^2+^]_i_-spike trains in agreement with the experimental observations only if BK channel densities are negligible. If one assumes that the unitary conductance of BK channels in marine invertebrates is closer to the estimated in mollusk and crustacean cells [40, 41, 42] (under asymmetric conditions that approach physiological gradients) than its counterpart in mammalian cells [43], this would be of the order of ≈70 pS. Under these conditions, the BK conductance densities that allow for oscillatory trains would imply channel counts of less than 1 molecule per flagellum, given the membrane area (Table 2). Therefore, although BK would play a key role in a scenario wherein Ca_V_ channels were the ones driving calcium, the role of this channel, if any, should be minimal if CatSper turns out to be the predominant channel. The role for BK in [Ca^2+^]_i_ responses to SAP was inferred indirectly by the alterations of the period of these oscillations by blockers such as niflumic acid [11, 44] and Iberiotoxin [17], which were corroborated by discrete logical network models. In addition, proteomic studies in sea urchin sperm have detected BK [18]. In the present quantitative framework, there are shared effects of BK both on the Ca_V_+BK and CatSper+NHE modules, such as defining a tonic-like minimal value for the nadir of [Ca^2+^]_i_ oscillations and increasing the period of the oscillations. This notwithstanding, the CatSper+NHE module precludes the presence of BK in sea urchin sperm at significant levels.

The use of models based on differential equations allowed us to confront their predictions with several quantitative data sets, gaining insights beyond those stemming from logical network models. The slow quantitative dynamics in cGMP as well as the trends in the spike amplitudes and interspike intervals of the [Ca^2+^]_i_-spike trains, which were instrumental in our analysis, are a kind of rich information source to which the discrete logical network modelling is blind. We also improved the continuous model of the [Ca^2+^]_i_ core oscillator in urchin sperm developed before [21], not only by bringing the network composition to the state of the art knowledge, but by grounding the analysis on experimental measurements at multiple levels of the network. Despite these new insights provided by ordinary differential equations, we foresee future directions for theoretical studies using other quantitative frameworks. The reports on the spatial localisation of CatSper channels in 4 distinct bands along the flagellum of both mouse [45] and human [46] sperm is fascinating and calls for partial differential equations to understand the significance of such topological organisation. In our models we assumed that molecular species such as cGMP, Ca^2+^ ions and protons were diluted uniformly within the volume of the flagellum. This likely imposed quantitative constraints on parameters that might be alleviated by considering local concentration asymmetries arising from the spatial arrangement of the membrane channels relative to each other (e.g. in human sperm Hv1 channels, the putative major pH_i_ regulator, are located between two of the CatSper bands [46]). In restricted domains, ample oscillations in ion concentrations may be enabled by smaller conductance densities than the ones required by our mean field model. Whether such a spatial arrangement of multiple channels exists in sea urchin sperm remains an intriguing question. The variance in the timing and amplitude of [Ca^2+^]_i_ spikes in individual cells, which was neglected in our analysis of the overall trends of the spike trains, may contain information on the underlying stochastic processes that could be better captured by Gillespie-type stochastic simulations or spatial-stochastic models.

We used bifurcation analysis of a subset of the differential equations of the full system as a heuristic tool to understand how the temporal evolution of cGMP activity affects the envelope and intervals between [Ca^2+^]_i_ spikes. It is important to bear in mind that the present analysis involves an asymptotic approximation, i.e. in our case *G* was set to a fixed value as a way to transform this variable into a parameter and quantify the amplitude and period of the stable limit cycles. The experimental corroboration of such analysis would require development of a cGMP-clamping technique, analogously to classic voltage-clamping experiments or synthetic biology approaches that seem presently unfeasible in sea urchin sperm.

We end by noting that the present work illustrates the added value of quantitative analysis which beyond offering a framework to characterise mechanisms overcoming experimental limitations, helps to identify the main elements controlling the system. Quantitative modelling allows to define guidelines for future experiments to assess the theoretical predictions, to identify key properties and testable quantities, and to prioritise these properties and quantities according to their impact and significance for the system.

## 4 Models and methods

### 4.1 The general modelling framework

Our strategy is to describe the dynamics of the SAP-activated signalling pathway in order to understand how the [Ca^2+^]_i_ fluctuations are controlled by different molecular components. A system of ordinary differential equations (ODE) describes the dynamics of the membrane potential and of the intraflagellar concentrations of cGMP, protons and Ca^2+^. These four essential variables depend on the activities of different flagellar channels and enzymes, the dynamics of which are also described by ODE (Sec. 4.2). The four variables are the main observables in the model and offer the means to assess its predictions by comparison with experimental data.

#### 4.1.1 Membrane potential

The dynamics of the membrane potential, *V*, is described with a Hodgkin-Huxley formalism [47]. The temporal derivative of *V* is defined as a sum of ion current densities normalised by the flagellum specific capacitance, according to Kirchoff’s law of charge conservation:

$$\begin{array}{c} \frac{dV}{dt}=-\frac{1}{C_{m}}(I_{L}+\sum_{i}I_{i}) \end{array}$$ (1)

Each current density, indexed by *i*, is associated to a given channel type to be chosen from a set of ion channels, i.e. *i* ∈ {kn, kc, cv, bk, cs}. The current terms *I*_*i*_ are defined according to Ohm’s law:

$$\begin{array}{c} I_{i}=g_{i}f_{i}^{o}\left(V-E_{i}\right) \end{array}$$ (2)

where the *E*_*i*_ is the reversal potential of the channel *i*, *g*_*i*_ is the channel-specific maximal conductance density defined as the product of the channel unitary conductance by the channel density in the flagellum membrane, and $f_{i}^{o}$ is the fraction of open channels conducting current. The aggregate parameter *g*_*i*_ is a convenient description of the conductance density given that for most channels in sea urchin spermatozoa, the unitary channel conductance, the channel density or both are unknown. The fraction $f_{i}^{o}$ is a variable whose dynamics reflects the complex regulation of channel gating. Eq 2 is therefore a general framework and channel specific details are presented individually in Sec. 4.2.

Following the Hodgkin-Huxley tradition, we define a leakage current term *I*_*L*_ = *g*_*L*_(*V* − *E*_*L*_) that ensures that the resting membrane potential $\hat{V}=E_{m}$ is asymptotically reached by imposing the following constraint on *E*_*L*_, given the parameters and variables *f*_*i*_ at equilibrium:

$$\begin{array}{c} E_{L}=E_{m}(1+\sum_{i}\frac{g_{i}}{g_{L}}\hat{f_{i}}E_{i}) \end{array}$$ (3)

The leakage current is interpreted as the net current arising from all the channels and transporters that are not explicitly described in the model.

As a remark on notation, in this article a hat-decorated variable $\hat{x}$ refers to the equilibrium value of the variable *x*.

#### 4.1.2 Cyclic nucleotide concentration

The concentrations of the nucleotides cGMP and cAMP increase in the sperm flagellum in response to SAP stimuli. These nucleotides affect the permeability of several nucleotide-gated ion channels, in turn changing membrane potential. Considering that cGMP is the nucleotide showing the highest changes after SAP binding and that the experimental release of its analogues in the flagellum is sufficient to produce a dose response that closely mimics the response elicited by the physiologic ligand, we restricted our analysis to this nucleotide, neglecting the eventual role of cAMP.

The intraflagellar concentration of cGMP is denoted by *G* and its dynamics described by the following differential equation:

$$\begin{array}{c} \frac{dG}{dt}=\sigma_{G}-\delta_{G}G+\theta_{G}\rho_{G} \end{array}$$ (4)

In this equation, it is assumed that the activities of general guanylate cyclases and phosphodiasterases lead to a constitutive turnover of cGMP, with a constant source *σ*_*G*_ and turnover rate *δ*_*G*_. In the absence of SAP signals, this basal turnover results in a cGMP at rest stationary concentration *G*_*r*_ = *σ*_*G*_/*δ*_*G*_. The basal turnover is assumed to be perturbed by the additional production of cGMP due to active SAP receptors, described by the term *ρ*_*G*_ (Sec. 4.2.1). A phosphodiesterase that impinges on sperm motility has been described in sea urchin [30]. Although its GAF domains could indicate allosteric regulation of catalytic activity by cGMP itself, the phosphodiesterase response to cGMP gave no hint of such nonlinearity, justifying the assumption of constant turnover rate *δ*_*G*_. The same report has shown that the potential regulation of the phosphodiesterase activity by pH [30], and this activity could be also regulated by [Ca^2+^]_i_ via calmodolin, as documented in other cellular systems and species [23]. Eq 4 neglects any potential effects of pH_i_ and [Ca^2+^]_i_ on phosphodiesterases, which is a simplifying assumption that proved to be reasonable by comparison with quantitative data.

The variable *G* was compared with measurements of total cGMP by radioimmunoassay in bulk cell experiments previously reported [22]. Since *G* represents the effective intraflagellar concentration of cGMP available to bind to KCNG channels, it was necessary to introduce a scaling factor for the term *ρ*_*G*_. This factor, *θ*_*G*_, consists of the product of flagellar volume (*v*_*f*_), cGMP buffering capacity in the flagellum (*B*_*G*_) and Avogadro constant (*N*_*A*_). In this way, only a fraction of the total cGMP synthesised by the active receptors participates in the signaling response and the *G* signal is kept within the nM sensitivity range reported for KCNG channels [48] (Sec. 4.2.2).

#### 4.1.3 Proton concentration

The permeability of some channels is affected by cytoplasmic concentration of protons. The intraflagellar proton concentration, denoted by *H*, is described by the following differential equation:

$$\begin{array}{c} \frac{dH}{dt}=\sigma_{H}-\delta_{H}H-\frac{J_{H}}{v_{f}} \end{array}$$ (5)

which assumes that there is a source of intraflagellar protons, *σ*_*H*_, a basal efflux rate *δ*_*H*_ and a variable efflux *J*_*H*_ mediated by the activity of the sodium/proton exchanger (Sec. 4.2.4). The parameter values were constrained by imposing the basal proton concentration to be 7.9 × 10^-8^ M (pH_i_ = 7.1, Table 2), obtained by solving *H* for equilibrium. The relative changes of the variable *H* in time were further compared with pH-sensitive, fluorescent probe signals measured in bulk populations [34] or in individual cells [32].

#### 4.1.4 Intraflagellar Ca^2+^ concentration

The intraflagellar Ca^2+^ concentration, represented by the variable *C*, is the main observable of our study. Its dynamics is described by the following differential equation:

$$\begin{array}{cc} \frac{dC}{dt} & =\sigma_{C}-\delta_{C}C-\frac{s_{f}}{2v_{f}F}\sum_{j}I_{j} \end{array}$$ (6)

with *j* ∈ {cv, cs}. Just as in the case of the previous components, we assume that different sources and sinks of intraflagellar Ca^2+^ result in a net constant influx rate *σ*_*C*_ and a basal efflux rate *δ*_*C*_. The [Ca^2+^]_i_ dynamics is further controlled by the activities of specific Ca^2+^ channels, described by the third term in the equation as the sum of ionic current densities scaled by the ratio of flagellum membrane area over the product of ion charge of a Ca^2+^ ion, flagellar volume and Faraday constant. The list of currents to be considered will depend on the scenario being analysed.

The variable *C* can be compared to measurements by Ca^2+^-sensitive fluorescent probes, either in individual cells or in populations [14, 9, 22]. Because the population measurements give only information about the trend of the envelope of the spike train, we relied on individual cell measurements for the analysis of the temporal structure and the amplitude of the Ca^2+^ spikes, as described in Sec. 4.4.

### 4.2 Signalling components and channels

In the following section, we will present the molecular components and channels that were implicated in SAP signal transduction and that are relevant for our study. For each of these components we will describe: i) the evidence for their role in SAP-signalling, ii) a mathematical model for its dynamics, iii) the reference parameter values and how they were obtained.

#### 4.2.1 SAP-receptor and guanylate cyclase

The binding kinetics of SAP to its respective sperm receptor has been studied in sea urchin species such as *L. pictus* [49] and *S. purpuratus* [50], whose spermatozoa respond to the decapeptide speract. The reported values for the association and dissociation rate constants for speract imply that the formation of a ligand-receptor complex is essentially irreversible during a chemotactic response, with an average life time of several hours. Since the time scale of the SAP response is of the order of seconds, for the sake of simplicity we neglected the dissociation rate. In order to explain the kinetic features of experimental cGMP data [22] we propose the following mechanism: once free SAP molecules S bind to free receptors R_F_, they go through several states in an irreversible manner, each with decreasing guanylate cyclase activity, namely high activity R_H_, low activity R_L_ and inactive R_I_ receptor forms. In the context of previous work, the molecular species R_H_ and R_L_ might be considered as equivalent to the phosphorylated and dephosphorylated forms of guanylate cyclase, respectively [51, 52, 53, 28]. Receptor activation mechanism is represented by the following kinetic diagram:

$$\begin{array}{c} R_{F}+S\overset{\;r_{1}\;}{\to }R_{H}\overset{\;r_{2}\;}{\to }R_{L}\overset{\;r_{3}\;}{\to }R_{I} \end{array}$$ (7)

which is translated in the following ordinary differential equations:

$$\frac{dS}{dt}=-r_{1}\theta_{R}SR_{F}$$ (8)

$$\frac{dR_{F}}{dt}=-r_{1}SR_{F}$$ (9)

$$\frac{dR_{H}}{dt}=r_{1}SR_{F}-r_{2}R_{H}$$ (10)

$$\frac{dR_{L}}{dt}=r_{2}R_{H}-r_{3}R_{L}$$ (11)

subject to the mass conservation relations:

$$R_{T}=R_{F}+R_{H}+R_{L}+R_{I}$$ (12)

$$S_{T}=S+\theta_{R}\left(R_{H}+R_{L}+R_{I}\right)$$ (13)

with *S* being the concentration of free SAP S and *R*_*F*_, *R*_*H*_ and *R*_*L*_ and *R*_*I*_ being the number of the R_F_, R_H_, R_L_ and R_I_ receptor forms per cell, respectively. *R*_*T*_ and *S*_*T*_ are constants denoting the total receptors per cell and SAP concentration, respectively.

As in other studies of soluble ligand-membrane receptor interactions [54], we describe the amounts of soluble SAP molecules *S* in molar concentration and those of the membrane receptor forms, *R*_*T*_, *R*_*F*_, *R*_*H*_, *R*_*L*_ and *R*_*I*_, in molecules per cell. As the SAP binds to the membrane receptors, its concentration in solution decreases proportionally to the bound receptor forms with a constant *θ*_*R*_, and this decrease is described by the second term of the conservation Eq 13, *θ*_*R*_(*R*_*H*_ + *R*_*L*_ + *R*_*I*_). This representation is convenient to model bulk studies as we set *θ*_*R*_ = *s*/*N*_*A*_, where *s* is the number of sperms per litre and *N*_*A*_ is the Avogadro constant.

Regarding the activation of guanylate cyclases stimulated by SAP, minor differences exist at the molecular level between sea urchin taxonomic orders. In sperm of *S. purpuratus* and *L. pictus*, which belong to the Echinoida order, receptor and guanylate cyclase have been reported to be two separate yet presumably tightly coupled membrane-bound proteins; once a SAP molecule binds to the former [55] this in turn stimulates the activity of the latter [56]. In the third species, which belongs to Arbacioida order, a single protein has the activities of ligand binding and guanylate cyclase [57, 58]. To capture the commonalities in a single model, we only take the latter and simpler case, thus ignoring any possible delay between SAP binding and guanylate cyclase activation. Thus, the term corresponding to the SAP-dependent cGMP synthesis, *σ*_*G*_, in Eq 4 is defined as:

$$\rho_{G}=k_{H}R_{H}+k_{L}R_{L}$$ (14)

where the constants *k*_*H*_ and *k*_*L*_ measure the guanylate cyclase activities of the high (*R*_*H*_) and low activity (*R*_*L*_) SAP-receptor forms, respectively.

#### 4.2.2 Cyclic nucleotide gated K^+^ channel

The K^+^-selective cyclic nucleotide gated (KCNG) channel is a pseudotetrameric channel with up to 4 cyclic nucleotide binding domains [59, 48], nonetheless, the binding of only a single cGMP molecule is necessary and sufficient to open a channel, thus giving a non-cooperative gating unlike most cyclic nucleotide gated channels [60, 48]. We therefore assume that the fraction of open channels, denoted as $f_{\text{kn}}^{o}$, increases with a rate directly proportional to the cGMP activity (*G*) and decreases with first order kinetics.

$$\begin{array}{c} \frac{df_{\text{kn}}^{o}}{dt}=\alpha_{\text{kn}}G\left(1-f_{\text{kn}}^{o}\right)-\beta_{\text{kn}}f_{\text{kn}}^{o} \end{array}$$ (15)

Expression for both the steady state fraction of open channels, ${\hat{f}}_{\text{kn}}^{o}$, and the characteristic time, *τ*_kn_, can be derived from the opening and closing rates:

$${\hat{f}}_{\text{kn}}^{o}=\frac{\alpha_{\text{kn}}G}{\alpha_{\text{kn}}G+\beta_{\text{kn}}}=\frac{G}{G+K_{\text{kn}}}$$ (16)

$$\tau_{\text{kn}}=\frac{1}{\alpha_{\text{kn}}G+\beta_{\text{kn}}}=\frac{1}{\alpha_{\text{kn}}\left(G+K_{\text{kn}}\right)}$$ (17)

with $K_{\text{kn}}=\frac{\beta_{\text{kn}}}{\alpha_{\text{kn}}}$. The ion current density through these channels is modelled as:

$$\begin{array}{c} I_{\text{kn}}=g_{\text{kn}}f_{\text{kn}}^{o}\left(V-E_{K}\right) \end{array}$$ (18)

where *E*_*K*_ is the Nernst potential of the K^+^ and *g*_kn_ denotes the maximal conductance density of the KCNG channel (the product of the unitary conductance by the density of channels).

#### 4.2.3 Hyperpolarisation-activated cyclic nucleotide-gated channels

Hyperpolarisation-activated cyclic nucleotide-gated channels from sea urchin sperm (spHCN) are cationic channels, weakly selective and carry a mixed current of Na^+^ and K^+^ with a permeability ratio *P*_K_/*P*_Na_ of ∼4-5 and a reversal potential of *E*_hc_ = -30 mV-−10 mV which would allow an inward Na^+^-current under physiological conditions that, when open, depolarises the cell membrane [61, 62, 63]. Unlike typical spHCN channels, spHCN-current inactivates in the absence of cAMP, and the latter increases the maximal open-probability instead of shifting the half-activation voltage of the G/V curve [62, 64]. Notwithstanding that, for the ionic current equation associated with this channel, we assumed in the model that cAMP is always present at sufficient concentration to avoid current inactivation, hence any role of cAMP in the overall dynamics is neglected. Following a classical Hodgkin-Huxley type model, we propose that spHCN opening depends on three independent gating variables, denoted by *m*_hc_, such that the fraction of open channels is $f_{\text{hc}}^{o}=m_{\text{hc}}^{3}$. The dynamics of the gating variables is described by:

$$\begin{array}{cc} \frac{dm_{\text{hc}}}{dt}= & \frac{1}{\tau_{\text{hc}}}({\hat{m}}_{\text{hc}}-m_{\text{hc}}) \end{array}$$ (19)

wherein the steady state fraction of active gating variable, ${\hat{m}}_{\text{hc}}$, is modelled with a Boltzmann equation, whereas the characteristic time, *τ*_hc_, is modelled as a Gaussian function of the voltage:

$$\begin{array}{cc} {\hat{m}}_{\text{hc}}= & \frac{1}{1+e^{\frac{V-h_{1}}{h_{2}}}} \end{array}$$ (20)

$$\tau_{\text{hc}}=h_{3}+h_{4}e^{-{\left(\frac{V-h_{5}}{h_{6}}\right)}^{2}}$$ (21)

The current via spHCN channels *I*_hc_ is given by:

$$I_{\text{hc}}=g_{\text{hc}}f_{\text{hc}}^{o}\left(V-E_{\text{hc}}\right)=g_{\text{hc}}m_{\text{hc}}^{3}\left(V-E_{\text{hc}}\right)$$ (22)

#### 4.24 Electroneutral sodium/proton exchanger

A sperm-specific electroneutral sodium/proton exchanger (NHE) is expressed in sperm flagella membrane [65]. Its activity is enhanced with hyperpolarised membrane potentials and inhibited with depolarisation. When active, it makes use of the Na^+^ concentration gradient between external and internal media to extrude protons by means of a Na^+^/H^+^ electroneutral exchange mechanism with a 1:1 stoichiometry, consequently increasing pH_i_ [66]. Even though NHE is not a channel, it is distinguished from the rest of NHE family members in having a voltage sensor domain that is homologous to that of voltage-gated ion channels [13], which might explain the observed voltage-dependent Na^+^/H^+^ exchange in sea urchin sperm in spite of being an electroneutral net exchange [67, 66, 68, 69]. We therefore model this voltage dependency as a regular gating mechanism similar to that of Hodgkin-Huxley like equations. The fraction of active NHE molecules, denoted $f_{\text{nh}}^{a}$, is described by:

$$\frac{df_{\text{nh}}^{a}}{dt}=\alpha_{\text{nh}}\left(1-f_{\text{nh}}^{a}\right)-\beta_{\text{nh}}f_{\text{nh}}^{a}$$ (23)

where the activation and inactivation rates are the following functions of the membrane potential:

$$\alpha_{\text{nh}}=n_{1}\frac{V-n_{2}}{e^{\frac{V-n_{2}}{n_{3}}}-1}$$ (24)

$$\beta_{\text{nh}}=-n_{4}\frac{V-n_{5}}{e^{-\frac{V-n_{5}}{n_{6}}}-1}$$ (25)

Proton flux as a consequence of exchange activity of NHE, designated *J*_*H*_ (mol s^−1^), is modelled with reversible, rapid-equilibrium, random order bi-bi kinetics [70]:

$$\begin{array}{c} J_{H}=f_{\text{nh}}^{a}J_{\text{max}}\frac{H{\left[{\text{Na}}^{+}\right]}_{\text{o}}-10^{-{\text{pH}}_{o}}{\left[{\text{Na}}^{+}\right]}_{\text{i}}}{H{\left[{\text{Na}}^{+}\right]}_{\text{o}}+K_{\text{Na}}\left(H+10^{-{\text{pH}}_{o}}\right)+10^{-{\text{pH}}_{o}}{\left[{\text{Na}}^{+}\right]}_{\text{i}}} \end{array}$$ (26)

where: *J*_max_ (mol s^−1^) is the maximal exchange rate; [Na^+^]_o_ and [Na^+^]_i_ are the extracellular and the intraflagellar concentrations of Na^+^, respectively; pH_o_ is the extracellular pH; and *K*_Na_ is the dissociation equilibrium constant for Na^+^.

#### 4.2.5 Voltage-gated calcium channels

There has been suggestive evidence for the involvement of classical voltage-gated calcium channels in the SAP-activated response, i.e. Low-Voltage-Activated [14, 10] and High-Voltage-Activated channels [16]. Here we focus on T-type calcium channels, which belong to the first class and are known by being associated to spiking activity in other cell types. We model their kinetics by the following diagram, in which illustrates that they transit irreversibly through three states, namely, inactive Ca_V_^i^, closed Ca_V_^c^ and open Ca_V_^o^:

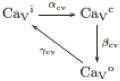


The gating rates are themselves voltage-dependent exponential functions similar to those of Hodgkin-Huxley (HH) type models:

$$\alpha_{\text{cv}}=v_{1}\frac{V-v_{2}}{e^{\frac{V-v_{2}}{v_{3}}}-1}$$ (27)

$$\beta_{\text{cv}}=-v_{4}\frac{V-v_{5}}{e^{-\frac{V-v_{5}}{v_{6}}}-1}$$ (28)

$$\gamma_{\text{cv}}=-v_{7}\frac{V-v_{8}}{e^{-\frac{V-v_{8}}{v_{9}}}-1}$$ (29)

Each transition rate is promoted at a different voltage range: recovery from inactivation (i to c) is favoured under hyperpolarisation (under the reference parameters we have *v*_2_ = −55 mV), inactivation (o to i) takes place under marked depolarisation (*v*_8_ = −18 mV) and opening of closed channels (c to o) is favoured from potentials slightly hyperpolarised (*v*_5_ = −39 mV).

Denoting the fraction of open, closed and inactive channels as $f_{\text{cv}}^{o}$, $f_{\text{cv}}^{c}$ and $f_{\text{cv}}^{i}$, respectively, we have two differential equations for the kinetics of the first two states, whereas the last one is calculated from the conservation equation $f_{\text{cv}}^{o}+f_{\text{cv}}^{c}+f_{\text{cv}}^{i}=1$.

$$\frac{df_{\text{cv}}^{o}}{dt}=\beta_{\text{cv}}f_{\text{cv}}^{c}-\gamma_{\text{cv}}f_{\text{cv}}^{o}$$ (30)

$$\frac{df_{\text{cv}}^{c}}{dt}=\alpha_{\text{cv}}\left(1-f_{\text{cv}}^{c}-f_{\text{cv}}^{o}\right)-\beta_{\text{cv}}f_{\text{cv}}^{c}$$ (31)

After solving gating equations for equilibrium, we get:

$$\begin{array}{cc} {\hat{f}}_{\text{cv}}^{o} & =\frac{\alpha_{\text{cv}}\beta_{\text{cv}}}{\beta_{\text{cv}}\gamma_{\text{cv}}+\alpha_{\text{cv}}\beta_{\text{cv}}+\gamma_{\text{cv}}\alpha_{\text{cv}}}\\ & =\frac{1}{1+\gamma_{\text{cv}}(\frac{1}{\alpha_{\text{cv}}}+\frac{1}{\beta_{\text{cv}}})} \end{array}$$ (32)

The ion current density through these channels is modelled as:

$$\begin{array}{c} I_{\text{cv}}=g_{\text{cv}}f_{\text{cv}}^{o}\left(V-E_{\text{Ca}}\right) \end{array}$$ (33)

#### 4.2.6 CatSper channels

The sea urchin genome contains the gene set necessary for the expression of the four core and the three auxiliary subunits that constitute the CatSper channel [71]. Furthermore, the expression of these subunits has been demonstrated in *A. punctulata* sperm flagella [12] and most of them in *S. purpuratus* [18]. In order to model CatSper gating, we consider three regulatory mechanisms reported in studies from mouse, human and sea urchin sperm: voltage-dependence, modulation of voltage-sensitivity by pH_i_, and inactivation by intracellular Ca^2+^ [72, 73, 74, 12]. We implement these mechanisms using two independent gating variables, *m*_cs_ and *h*_cs_, representing the fraction of channels with their gate modulated by both voltage and pH_i_ open, and the fraction of channels with Ca^2+^-dependent gate that is not inactivated by calcium. The fraction of open CatSper channels conducting Ca^2+^ currents, $f_{\text{cs}}^{o}$, is given by the product of these gating variables:

$$\begin{array}{c} f_{\text{cs}}^{o}=m_{\text{cs}}h_{\text{cs}} \end{array}$$ (34)

whose dynamics is described by the following differential equations:

$$\frac{dm_{\text{cs}}}{dt}=\alpha_{m}\left(1-m_{\text{cs}}\right)-\beta_{m}m_{\text{cs}}$$ (35)

$$\frac{dh_{\text{cs}}}{dt}=\alpha_{h}\left(1-h_{\text{cs}}\right)-\beta_{h}h_{\text{cs}}$$ (36)

The opening and closing rates of the voltage- and pH-dependent gate were chosen to be the following symmetric functions:

$$\alpha_{m}=s_{1}e^{-\frac{v_{\text{cs}}-V}{2s_{2}}}$$ (37)

$$\beta_{m}=s_{1}e^{\frac{v_{\text{cs}}-V}{2s_{2}}}$$ (38)

such that the equilibrium open gate curve is a Boltzmann function of the voltage:

$${\hat{m}}_{\text{cs}}=\frac{\alpha_{m}}{\alpha_{m}+\beta_{m}}=\frac{1}{1+e^{\frac{v_{\text{cs}}-V}{s_{2}}}}$$ (39)

The dependence on pH_i_ is ensured by defining the half-maximal voltage, *v*_cs_, as the following function of the proton concentration *H*:

$$v_{\text{cs}}=s_{3}\frac{s_{4}^{s_{5}}}{{\left(-{\text{log}}_{10}H\right)}^{s_{5}}+s_{4}^{s_{5}}}+s_{6}$$ (40)

in order to produce a shift of the gating curve towards more negative membrane potentials upon alkalinisation of cytosol, as observed in [72, 73, 12].

The second gate, inhibited by [Ca^2+^]_i_, is assumed to switch between open and closes states with the following rates:

$$\alpha_{h}=s_{10}{\left(s_{8}/s_{7}\right)}^{s_{9}}$$ (41)

$$\beta_{h}=s_{10}{\left(C/s_{7}\right)}^{s_{9}}$$ (42)

whose function forms were chosen such that when solving for the open gate fraction at equilibrium, ${\hat{h}}_{\text{cs}}$, one obtains a Hill-function:

$${\hat{h}}_{\text{cs}}=\frac{\alpha_{h}}{\alpha_{h}+\beta_{h}}=\frac{{s_{8}}^{s_{9}}}{s_{8}^{s_{9}}+C^{s_{9}}}$$ (43)

The ion current density through CatSper is modelled as:

$$I_{\text{cs}}=g_{\text{cs}}f_{\text{cs}}^{o}\left(V-E_{\text{Ca}}\right).$$ (44)

#### 4.2.7 Big-conductance potassium channels

Big-conductance potassium channels (BK, also known as Slo1) are activated by [Ca^2+^]_i_. These channels have been implicated in sea urchin sperm signalling by pharmacological blocking assays [11, 17] and adopted in discrete models [17, 20, 18]. For their dynamics we assume two states, open or closed, where the open-channel fraction, denoted by $f_{\text{bk}}^{o}$, is described by the following differential equation:

$$\frac{df_{\text{bk}}^{o}}{dt}=\alpha_{\text{bk}}\left(1-f_{\text{bk}}^{o}\right)-\beta_{\text{bk}}f_{\text{bk}}^{o}$$ (45)

with the closing rate kept as a constant *β*_bk_ and the opening rate being the following Ca^2+^-dependent function:

$$\alpha_{\text{bk}}=b_{1}\frac{b_{2}-C}{e^{\frac{b_{2}-C}{b_{3}}}-1}$$ (46)

The ion current density through these channels is expressed as:

$$I_{\text{bk}}=g_{\text{bk}}f_{\text{bk}}^{o}\left(V-E_{K}\right)$$ (47)

### 4.3 Computational implementation and parameter estimations

The systems of differential equations were solved numerically with Mathematica 11 (Wolfram Research, Inc., Mathematica, Version 11.0, Champaign, IL), XPPAUT 8 [24] or R (using the package deSolve [75]). XPPAUT 8 was used for the bifurcation analysis (see below). Mathematica was further used to fit the model to experimental data using the Nonlinearmodelfit function with the optimisation method Differential Evolution with constraints. In the analysis, we defined several model variants based on subsets of the variables and equations presented above. Some of these defined modules were used to estimate the parameters and some were used to analyse specific properties.

The Upstream module (illustrated in Fig 1) was fitted to data in two phases:

- The state space comprised by the variables {*S*, *R*_*F*_, *R*_*H*_, *R*_*L*_, *G*}, which includes the reactions involving the SAP-receptor (Eqs 8–11) and the cGMP dynamics (Eq 4), was fitted to kinetic data of cGMP measured in bulk cell populations [22]. Data points were read from the indicated publication and are expressed in units of pmol per 10^8^ cells. The cGMP kinetics obtained with the different SAP concentrations used in [22] were fitted simultaneously with the model (S1 Fig). During this fitting the dimension cGMP was pmol per 10^8^ cells instead of molar concentration, such that the scaling factor *θ*_*G*_ (described in the Sec. 4.1.2) was not called in. The parameter value of *σ*_*G*_ obtained in this phase, being measured in pmol per 10^8^ cells per s, was subsequently rescaled to molar concentrations per s, taking into account the flagellar volume.
- The remaining variables of the module ($f_{\text{kn}}^{o}$, $f_{\text{hc}}^{o}$, *V*), which involve the early electrophysiological changes caused by SAP (i.e. hyperpolarisation and repolarisation due to the opening of KCNG and spHCN, respectively), were used to fit the gating parameters of the ion channels. The spHCN parameters were either extracted or calculated from [62, 63, 61, 76], while those of KCNG were based on the values reported in [10, 59, 48]. Fixing these values, the conductance density parameters of these channels were calibrated together with *B*_*G*_ (included as a free parameter in the scaling factor *θ*_*G*_), by fitting the dynamics of *V* with the kinetic data from [10], which comprise the early response of the membrane potential change. Only the first second of the time series was used in the fitting, taking into consideration that, by itself, this module is expected to fit only the early transient behaviour of *V* before other downstream channels with strong effect come into play.

In order to assess and calibrate this module, we assumed that the overall dynamics under both single-cell and population regimes can be compared using a scaling factor; this depends on the flagellar volume term to convert the cGMP signal into the effective concentration capable of exerting a physiological effect at the single cell level (see parameter *θ*_*G*_, Sec. 4.1.2).

To study the dynamics of intraflagellar calcium, we set out to explore the possible contribution of different channels under two main scenarios.

- In one scenario, the model features the Ca_V_+BK module and has the state space {*S*, *R*_*F*_, *R*_*H*_, *R*_*L*_, *G*, $f_{\text{kn}}^{o}$, $f_{\text{hc}}^{o}$, *V*, $f_{\text{cv}}^{o}$, $f_{\text{cv}}^{c}$, $f_{\text{bk}}^{o}$, *C*}. The values of the Ca_V_ channel parameters were adjusted based on the characteristics of low-voltage-activated T-type Ca^2+^ channels [77], for which there is evidence suggesting their presence in sperm [14, 10] (Table 1).
- In the alternative scenario, the model features the CatSper+NHE module and has the following state space {*S*, *R*_*F*_, *R*_*H*_, *R*_*L*_, *G*, $f_{\text{kn}}^{o}$, $f_{\text{hc}}^{o}$, *V*, *m*_cs_, *h*_cs_, $f_{\text{nh}}^{a}$, *C*, *H*}. The CatSper gating parameters were set such that its [Ca^2+^]_i_ and pH_i_ sensitivity range is placed within the physiologically expected values in reported natural SAP responses (S3 Fig).

The channel parameters were tuned such that the numerical solution of the variable *C* representing [Ca^2+^]_i_ in the model were comparable to those of the experimental time series of Ca^2+^-sensitive fluorescent probes using the criteria defined in the next Subsection 4.4.

Finally, to understand how the Ca^2+^-spike train patterns are controlled by the Ca_V_+BK and the CatSper+NHE module, we performed bifurcation analyses of two simpler models, in which *G* was input parameter and state spaces were respectively $\{f_{\text{kn}}^{o}$, $f_{\text{hc}}^{o}$, *V*, $f_{\text{cv}}^{o}$, $f_{\text{cv}}^{c}$, $f_{\text{bk}}^{o}$, *C*} and $\{f_{\text{kn}}^{o}$, $f_{\text{hc}}^{o}$, *V*, *m*_cs_, *h*_cs_, $f_{\text{nh}}^{a}$, *C*, *H*}.

### 4.4 Comparison of the structure of the Ca-spike trains in experiments and models

The [Ca^2+^]_i_ dynamics in response to SAP was imaged in individual *S. purpuratus* sperm immobilised on a cover slip using intracellular Ca^2+^-sensitive fluorescent probe following the uncaging of a photoactivatable Speract analogue by a 0.3s pulse of UV light (experimental details in [78]). By measuring the average fluorescence of the flagellum of each cell at each time point we obtained a time series of this proxy of [Ca^2+^]_i_). Each time series was smoothed using a cubic spline (as implemented by the R function smooth.spline) and the maxima of the smooth curve used to identify Ca-spike trains that were characterised as (*t*_*k*_, *A*_*k*_, *T*_*k*_), where *k* is the ordered spike index (*k* = 1, 2, 3, …) and *t*_*k*_, *A*_*k*_, and *T*_*k*_ = *t*_*k*+1_ − *t*_*k*_ are the time, the amplitude and interspike interval. To summarise the structure of each individual time series we computed the average interspike time *T*_*k*_, and the regression coefficients of the relative amplitude *A*_*k*_/*A*_1_ and relative interspike interval *T*_*k*_/*T*_1_ over the spike index, denoted *b*_*A*_ and *b*_*T*_, respectively. To compare the [Ca^2+^]_i_ dynamics in the model the same procedure was applied to the numerical solutions of *C*. The structure of the spike trains in the model was considered to be coherent with those experimentally observed according to the following selection criteria: the average spike interval is in the range 0.37 s-1.38 s, the predicted (*b*_*T*_, *b*_*A*_) point falls within the convex hull defined by experimental values in the bottom right quadrant, i.e. the spike amplitude decrease whereas the interspike interval increase in time *b*_*A*_ < 0 and *b*_*T*_ > 0. As mentioned in the Sec. 2.3, this pattern of the spike trains is the most representative of the experimental time series and the most challenging for the models. In addition, to filter out spontaneous oscillations and alternative equilibria in multistable regimes, we imposed that the resting steady state value of [Ca^2+^]_i_, $\hat{C}$, obtained following numerical solutions with *S* = 0, is about 100nM. It is important to notice that the fluorescent probe intensities were not calibrated and therefore mean flagellar intensities are not accurate [Ca^2+^]_i_ measurements. We assumed that the fluorescence intensity was proportional to the actual concentration of [Ca^2+^]_i_, focusing our analyses on relative differences and values. This assumption underlies the co-plot time series and model solutions presented in the figures and also the direct comparison of the theoretical and experimental *b*_*A*_ values. We believe this assumption is reasonable, considering that all Ca-fluorescence intensity spikes analysed were below the control intensities elicited by ionomycin.

### 4.5 Mixed scenarios parametrisation and parameter sensitivity

We analysed mixtures of Ca_V_+BK and CatSper+NHE modules by assembling the most extended model with state space {*S*, *R*_*H*_, *R*_*L*_, *G*, $f_{\text{kn}}^{o}$, $f_{\text{hc}}^{o}$, *V*, $f_{\text{cv}}^{o}$, $f_{\text{cv}}^{c}$, $f_{\text{bk}}^{o}$, *m*_cs_, *h*_cs_, $f_{\text{nh}}^{a}$, *C*, *H*}. The parameters of this model are the union of all the parameters listed in Table 2.

Any mixed scenario involving the two modules can be parameterised by setting:

$$g_{\text{cv}}=\theta^{\text{cv}}g_{\text{bk}}^{\text{cv}}$$ (48)

$$g_{\text{bk}}=\theta^{\text{cv}}g_{\text{bk}}^{\text{cv}}$$ (49)

$$g_{cs}=\theta^{\text{cs}}g_{\text{cs}}^{\text{cs}}$$ (50)

$$g_{L}=\theta^{\text{cv}}g_{L}^{\text{cv}}+\theta^{\text{cs}}g_{L}^{\text{cs}}$$ (51)

$$\delta_{C}=\theta^{\text{cv}}\delta_{C}^{\text{cv}}+\theta^{\text{cs}}\delta_{C}^{\text{cs}}$$ (52)

with the superscripts cv and cs denoting the reference values in the Ca_V_+BK and the CatSper+NHE modules, respectively, and all the remaining parameters taking the values in Table 2. *θ*^cv^ and *θ*^cs^ are non-dimensional scaling factors that allow to weigh the contribution of each module. With this formulation, the original Ca_V_+BK module dynamics can be recovered in this extended model by setting *θ*^cv^ = 1 and *θ*^cs^ = 0, whereas the CatSper+NHE module can be obtained with *θ*^cv^ = 0 and *θ*^cs^ = 1.

From the above equations, we can express a ratio of the maximal conductance density of CatSper with respect to that of Ca_V_:

$$r_{g}=\frac{\theta^{\text{cs}}g_{\text{cs}}}{\theta^{\text{cv}}g_{\text{cv}}}$$ (53)

In a first approach to mixed scenarios we interpolated between the two modules. Starting with the CatSper+NHE module we added fractions of Ca_V_ and BK channels in three ways: a) adding Ca_V_ + BK while decreasing proportionally CatSper by setting *θ*^cv^ = *θ* and *θ*^cs^ = (1 − *θ*) with *θ* ∈ [0, 1] (S4 Fig); b) adding only Ca_V_ by setting *θ*^cv^ = *θ* and *θ*^cs^ = (1 − *θ*) while forcing *g*_bk_ = 0 (S5A Fig); and c) adding only BK by defining $g_{\text{bk}}=\theta g_{\text{bk}}^{\text{cv}}$ and setting *θ*^cv^ = 0 and *θ*^cs^ = 1 (S5B Fig).

A more systematic analysis of mixed scenarios contemplated the conductance of channels that compose the two modules but also variants of the values of all the other parameters, except for those representing the Upstream Module (which were fixed at their point estimates in Table 2). The exploration of this parameter hyperspace was done using an algorithm inspired on adaptive evolutionary dynamics, specified in S1 Algorithm. Briefly, the algorithm comprehends two phases, one of neutral exploration of the parameter space by evolving a seed population of parameter vectors in a regular grid, followed by further exploitation of parameter vectors selected according to the criteria described above (Sec. 4.4). First, each parameter vector $\vec{\lambda }$ in the nodes of the grid was obtained by taking *θ*^cv^, *θ*^cs^ ∈ {0.0001, 0.1, 0.2, 0.3, …, 2.0}. Each vector in this population was used randomly as a seed to generate a daughter parameter vector obtained by multiplying each parameter value by an independent lognormal distributed random variable (specified as *e*^*x*^ with *x* being normally distributed with mean 0 and standard deviation 0.04). Each daughter parameter vector was appended to the evolving population, irrespective of the output behaviour, serving as random seed to generate new daughters. The method to generate parameter vector variants favoured values close to the reference ones (Table 2), reducing the probability that the random exploration would spread to unrealistic regions of the parameter space (e.g. leading to inactivation, opening and closing of Ca_V_ within incongruous membrane potential intervals). The dynamics predicted by each parameter vector $\vec{\lambda }$, seeds and daughters alike, was obtained by solving numerically the extended model. The process of generation of new parameter vectors was repeated until the clouds of points, spreading from the nodes of the grid, once projected in the plane (*g*_*cv*_, *g*_*cs*_), started to visibly coalesce. At this stage, several parameter vectors satisfied the selection criteria for coherence with experimental observations and were selected for further exploration in the second phase of the algorithm. In each iteration, a vector in the selected population was randomly picked to generate a daughter vector by the same procedure as above. This daughter vector was analysed and added to the evolving selected population, serving as seed for further exploration, if it met the selection criteria; otherwise it was stored in a list of unselected parameter vectors. The latter vectors are by construction closely located in the parameter space to those of the evolving selected population from which they derived but, being incoherent with observations, they were not further explored. The selected and unselected vector populations were used to build clusters based on the proximity in the projection plane (*g*_*cv*_, *g*_*cs*_). The iterative generation of daughter parameters from the selected population was repeated until the clustering in the plane (*g*_*cv*_, *g*_*cs*_) was stable (i.e. no new clusters were observed after a large number of iterations). Within each final cluster, the fraction of number of selected parameter vectors over the total number of vectors is a kind of measure of the parameter sensitivity. The smaller this fraction the more sensitive the model is within the cluster.

### 4.6 Resting state and initial conditions

Initial conditions of the gating variables of KCNG, spHCN, Ca_V_, BK and NHE, as well as parameters *σ*_*C*_, *E*_*L*_ and *δ*_*H*_, were all calculated by solving the system at equilibrium in the absence of SAP, that is, setting each differential equation to 0 under the condition *S* = 0. Physiological observables *G*, *C*, *H* and *V* were initialised to their respective resting values, as reported in the literature (Table 2).

The parameters governing NHE activation were chosen such that approximately 40% of exchangers were active at the resting state, as inferred from experimental data in activated sperm without any chemoattractant stimulation [35, 79], whereas the maximum effective proton flux by NHE, *J*_max_, was fitted to limit the pH_i_ up to ∼7.7, after physiological stimulation by SAP [80, 81].

## Supporting information

S1 Fig Kinetics of intracellular cGMP concentration elicited by different SAP concentrations in bulk sperm populations and its modelling. The graph shows the time courses of cGMP concentration elicited by the indicated SAP concentrations (values in nM). For each SAP concentration (encoded in grey shades) the dots represent experimental values read from the figures in [22] and the lines are the single cell numerical solutions of the variable *G* in the Upstream module, with state space {*S*, *R*_*F*_, *R*_*H*_, *R*_*L*_, *G*} that best fit the ensemble of the data (i.e. the four curves simultaneously). The values of *R*_*T*_, *r*_2_, *r*_3_, *δ*_*G*_, *k*_*L*_ and *k*_*H*_ were fitted while fixing the remaining parameters (Table 2). Because the measurements of the SAP-receptor association rate constant *r*_1_ are technically more reliable than those of the number of receptors *R*_*T*_ (reported values include 14000 [88, 22], 3 × 10^5^ [28] and 1 × 10^6^ [27] molecules per cell), we fixed the value of *r*_1_ and fitted *R*_*T*_. We fixed also the value of the factor that converts the amount receptor-bound SAP molecules to soluble concentration *θ*_*R*_ = *s*/*N*_*A*_ = 6.14 × 10^−5^ nMcell based on the reported cell density *s* = 3.7 × 10^7^ cellmL^−1^ [22]. Finally, *G*_*r*_ was preset to 1.24 nM [22] and the condition *σ*_*G*_ = *G*_*r*_/*δ*_*G*_ held during the fitting.
(TIF)

S2 Fig Calibration of spHCN gating parameters. In the upper panel, experimental traces of ionic currents measured by whole cell patch clamp technique in HEK cells expressing heterologously spHCN and loaded with photoactivatable cAMP analog, which in turn was uncaged by UV light. The set of currents correspond to different voltage pulses (indicated at the end of the trace). Data extracted from figure 4a of [62] (gray lines). Each trace was fitted to an an exponential function (black dashed lines) in order to estimate the characteristic activation time (*τ*). In the lower pannel, the set of estimated characteristic times was fitted to a Gaussian function, which is our proposed form for the voltage-dependent characteristic time of spHCN gating (Eqs 20 and 21).
(TIF)

S3 FigCalibration of pH- and voltage-dependent gate of the CatSper channel.In A and B, experimental data on mouse sperm (circles) are shown, along with model fittings (lines) related to the voltage- and pH_i_-dependent gate variable in equilibrium, ${\hat{m}}_{\text{cs}}$. Model fittings in mouse and sea urchin cases are plotted with dashed and bold lines, respectively. The experimental data of A correspond to the current amplitude of divalent ions (symmetrical Ba^2+^) produced by taking the holding voltage from 0 mV to −100 mV, under different pH_i_ values, and measured by whole-cell patch-clamp in mouse sperm, (data extracted from Fig. 4c of [72]). Mouse data displayed in A and B were simultaneously fitted to the Eqs 44 and 40. To calibrate sea urchin’s ${\hat{m}}_{\text{cs}}$, we took the parameters obtained with mouse data as a starting point and manually adjusted them, constraining that the pH_i_ sensitivity should be within the physiological pH_i_ response observed in sea urchin sperm (area marked in gray). C and D correspond to the G/V (conductance/voltage) curves of mouse and sea urchin CatSper, respectively, using the Eq 39. In C, the parameters reported in [72] were used). In panel D, the resting membrane potential is indicated with a gray dashed line as reference. Taking into account that the S4 segment of CatSper voltage sensor domain has more positive charges in the sea urchin homologue protein that in the mammalian counterparts [12], we envisioned that its voltage sensitivity should be steeper. Thus, a 3-fold decrease was introduced to the voltage sensitivity parameter, *s*_2_, as an initial guess. This last parameter has not been estimated in sea urchin due to the lack of patch-clamp measurements.(TIF)

S4 Fig Titration of the module Ca_V_ + BK on the CatSper module. Starting from the model that includes the CatSper module, numerical solutions for calcium are shown under different values of the weighting parameter *θ*, which controls the percentage of module Ca_V_ + BK that is being added. The parameters are set according to Eqs 48–52 with *θ*^cv^ = *θ* and *θ*^cs^ = (1 − *θ*). For reference, the original CatSper-only scenario is shown first, i.e. *θ* = 0, and the subsequent rows correspond to the gradual increase of *θ*. The effect of this parameter on the channel densities is reported in the coefficient *r*_*g*_, which measures the total conductance ratio CatSper/Ca_V_, as well as the corresponding *g*_bk_ value. The greater the value of *r*_*g*_, the greater the predominance of CatSper on the total calcium conductance with respect to Ca_V_.
(TIFF)

S5 Fig Titration of Ca_V_ or BK channels on the CatSper module. In A and B, we show numerical solutions for calcium under different values of the weighting parameter *θ*, with either BK or CaV conductance density set to 0, respectively. As reference, the original scenario of only CatSper, i.e. *θ* = 0, is shown in the top row, while the subsequent rows correspond to the gradual increase of *θ*. In A, the reference value of the Ca_V_ conductance density, *g*_cv_, is multiplied directly by *θ*, whereas the reference value of CatSper conductance density is multiplied by (1—*θ*); The resulting ratio of CatSper/Ca_V_ conductance densities, *r*_*g*_, is shown for each *θ* value. In B, *θ* sets the fraction of the reference value of *g*_bk_ by multiplying the conductance density of BK; unlike scenario A, the conductance density of CatSper is not weighted by *θ*. For each titration, the corresponding modified *g*_bk_ value is shown.
(TIFF)

S1 Algorithm Two-phase parameter space exploration algorithm. (PDF)

## References

[1] Berridge MJ, Lipp P, Bootman MD. The versatility and universality of calcium signalling. Nature reviews Molecular cell biology. 2000;1(1):11–21. 10.1038/35036035 11413485

[2] Berridge MJ. Inositol trisphosphate and calcium signalling mechanisms. Biochimica et Biophysica Acta—Molecular Cell Research. 2009;1793(6):933–940. 10.1016/j.bbamcr.2008.10.005 19010359

[3] Goldbeter A, Dupont G, Berridge MJ. Minimal model for signal-induced Ca2+ oscillations and for their frequency encoding through protein phosphorylation. Proceedings of the National Academy of Sciences of the United States of America. 1990;87(4):1461–5. 10.1073/pnas.87.4.1461 2304911 PMC53495

[4] Díaz J, Pastor N, Martínez-Mekler G. Role of a spatial distribution of IP3 receptors in the Ca2+ dynamics of the Xenopus embryo at the mid-blastula transition stage. Developmental dynamics: an official publication of the American Association of Anatomists. 2005;232(2):301–12. 10.1002/dvdy.20238 15614769

[5] Menini A. Calcium signalling and regulation in olfactory neurons. Current Opinion in Neurobiology. 1999;9(4):419–426. 10.1016/S0959-4388(99)80063-4 10448159

[6] Kaupp UB, Seifert R. Cyclic nucleotide-gated ion channels. Physiological reviews. 2002;82(3):769–824. 10.1152/physrev.00008.2002 12087135

[7] Darszon A, Guerrero A, Galindo BE, Nishigaki T, Wood CD. Sperm-activating peptides in the regulation of ion fluxes, signal transduction and motility. The International journal of developmental biology. 2008;52(5-6):595–606. 10.1387/ijdb.072550ad 18649273

[8] Kaupp UB, Strünker T. Signaling in Sperm: More Different than Similar. Trends in Cell Biology. 2017;27(2):101–109. 10.1016/j.tcb.2016.10.002 27825709

[9] Böhmer M, Van Q, Weyand I, Hagen V, Beyermann M, Matsumoto M, et al Ca2+ spikes in the flagellum control chemotactic behavior of sperm. The EMBO journal. 2005;24(15):2741–52. 10.1038/sj.emboj.7600744 16001082 PMC1182239

[10] Strünker T, Weyand I, Bönigk W, Van Q, Loogen A, Brown JE, et al A K+-selective cGMP-gated ion channel controls chemosensation of sperm. Nature cell biology. 2006;8(10):1149–54. 10.1038/ncb1473 16964244

[11] Wood CD, Nishigaki T, Tatsu Y, Yumoto N, Baba Sa, Whitaker M, et al Altering the speract-induced ion permeability changes that generate flagellar Ca2+ spikes regulates their kinetics and sea urchin sperm motility. Developmental Biology. 2007;306(2):525–537. 10.1016/j.ydbio.2007.03.036 17467684

[12] Seifert R, Flick M, Bonigk W, Alvarez L, Trotschel C, Poetsch A, et al The CatSper channel controls chemosensation in sea urchin sperm. The EMBO Journal. 2014;34:379–392. 10.15252/embj.201489376 25535245 PMC4339123

[13] Wang D, King SM, Quill Ta, Doolittle LK, Garbers DL. A new sperm-specific Na+/H+ exchanger required for sperm motility and fertility. Nature cell biology. 2003;5(12):1117–22. 10.1038/ncb1072 14634667

[14] Wood CD, Darszon A, Whitaker M. Speract induces calcium oscillations in the sperm tail. The Journal of cell biology. 2003;161(1):89–101. 10.1083/jcb.200212053 12695500 PMC2172867

[15] Wood CD, Nishigaki T, Furuta T, Baba SA, Darszon A. Real-time analysis of the role of Ca(2+) in flagellar movement and motility in single sea urchin sperm. The Journal of cell biology. 2005;169(5):725–31. 10.1083/jcb.200411001 15928204 PMC2171626

[16] Granados-Gonzalez G, Mendoza-Lujambio I, Rodriguez E, Galindo BE, Beltrán C, Darszon A. Identification of voltage-dependent Ca2+ channels in sea urchin sperm. FEBS letters. 2005;579(29):6667–72. 10.1016/j.febslet.2005.10.035 16307742

[17] Espinal J, Aldana M, Guerrero A, Wood C, Darszon A, Martínez-Mekler G. Discrete dynamics model for the speract-activated Ca2+ signaling network relevant to sperm motility. PloS one. 2011;6(8):e22619 10.1371/journal.pone.0022619 21857937 PMC3156703

[18] Espinal-Enríquez J, Priego-Espinosa DA, Darszon A, Beltrán C, Martínez-Mekler G. Network model predicts that CatSper is the main Ca2+channel in the regulation of sea urchin sperm motility. Scientific reports. 2017;7(1):4236 10.1038/s41598-017-03857-9 28652586 PMC5484689

[19] Lishko PV, Mannowetz N. CatSper: a unique calcium channel of the sperm flagellum. Current Opinion in Physiology. 2018;2:109–113. 10.1016/j.cophys.2018.02.004 29707693 PMC5914511

[20] Espinal-Enríquez J, Darszon A, Guerrero A, Martínez-Mekler G. In silico determination of the effect of multi-target drugs on calcium dynamics signaling network underlying sea urchin spermatozoa motility. PloS one. 2014;9(8):e104451 10.1371/journal.pone.0104451 25162222 PMC4146467

[21] Aguilera LU, Galindo BE, Sánchez D, Santillán M. What is the core oscillator in the speract-activated pathway of the Strongylocentrotus purpuratus sperm flagellum? Biophysical journal. 2012;102(11):2481–2488. 10.1016/j.bpj.2012.03.075 22713563 PMC3368143

[22] Kaupp UB, Solzin J, Hildebrand E, Brown JE, Helbig A, Hagen V, et al The signal flow and motor response controling chemotaxis of sea urchin sperm. Nature cell biology. 2003;5(2):109–17. 10.1038/ncb915 12563276

[23] Francis SH, Blount MA, Corbin JD. Mammalian Cyclic Nucleotide Phosphodiesterases: Molecular Mechanisms and Physiological Functions. Physiological Reviews. 2011;91(2):651–690. 10.1152/physrev.00030.2010 21527734

[24] Ermentrout B. Simulating, Analyzing, and Animating Dynamical Systems. Society for Industrial and Applied Mathematics; 2002 Available from: http://epubs.siam.org/doi/book/10.1137/1.9780898718195.

[25] Romano DR, Pharris MC, Patel NM, Kinzer-Ursem TL. Competitive tuning: Competition’s role in setting the frequency-dependence of Ca^2+^-dependent proteins. PLOS Computational Biology. 2017;13(11):e1005820 10.1371/journal.pcbi.1005820 29107982 PMC5690689

[26] Faro J, von Haeften B, Gardner R, Faro E. A Sensitivity Analysis Comparison of Three Models for the Dynamics of Germinal Centers. Frontiers in Immunology. 2019;10 10.3389/fimmu.2019.02038 31543878 PMC6729701

[27] Kashikar ND, Alvarez L, Seifert R, Gregor I, Jäckle O, Beyermann M, et al Temporal sampling, resetting, and adaptation orchestrate gradient sensing in sperm. The Journal of cell biology. 2012;198(6):1075–91. 10.1083/jcb.201204024 22986497 PMC3444779

[28] Pichlo M, Bungert-Plümke S, Weyand I, Seifert R, Bönigk W, Strünker T, et al High density and ligand affinity confer ultrasensitive signal detection by a guanylyl cyclase chemoreceptor. The Journal of cell biology. 2014;206(4):541–57. 10.1083/jcb.201402027 25135936 PMC4137060

[29] Jikeli JF, Alvarez L, Friedrich BM, Wilson LG, Pascal R, Colin R, et al Sperm navigation along helical paths in 3D chemoattractant landscapes. Nature communications. 2015;6:7985 10.1038/ncomms8985 26278469 PMC4557273

[30] Su YH, Vacquier VD. Cyclic GMP-specific phosphodiesterase-5 regulates motility of sea urchin spermatozoa. Molecular biology of the cell. 2006;17(1):114–21. 10.1091/mbc.E05-08-0820 16236790 PMC1345651

[31] Alvarez L, Dai L, Friedrich BM, Kashikar ND, Gregor I, Pascal R, et al The rate of change in Ca2+ concentration controls sperm chemotaxis. The Journal of cell biology. 2012;196(5):653–63. 10.1083/jcb.201106096 22371558 PMC3307702

[32] González-Cota AL, Silva PÂ, Carneiro J, Darszon A. Single cell imaging reveals that the motility regulator speract induces a flagellar alkalinization that precedes and is independent of Ca2+ influx in sea urchin spermatozoa. FEBS letters. 2015;589(16):2146–54. 10.1016/j.febslet.2015.06.024 26143372

[33] Feijó JA, Sainhas J, Holdaway-Clarke T, Cordeiro MS, Kunkel JG, Hepler PK. Cellular oscillations and the regulation of growth: the pollen tube paradigm. BioEssays: news and reviews in molecular, cellular and developmental biology. 2001;23(1):86–94. 10.1002/1521-1878(200101)23:1<86::AID-BIES1011>3.0.CO;2-D 11135313

[34] Nishigaki T, Wood CD, Tatsu Y, Yumoto N, Furuta T, Elias D, et al A sea urchin egg jelly peptide induces a cGMP-mediated decrease in sperm intracellular Ca 2+ before its increase. Developmental Biology. 2004;272(2):376–388. 10.1016/j.ydbio.2004.04.035 15282155

[35] Christen R, Schackmann RW, Shapiro BM. Elevation of the intracellular pH activates respiration and motility of sperm of the sea urchin, Strongylocentrotus purpuratus. The Journal of biological chemistry. 1982;257(24):14881–90. 7174670

[36] Christen R, Schackmann RW, Shapiro BM. Metabolism of sea urchin sperm. Interrelationships between intracellular pH, ATPase activity, and mitochondrial respiration. The Journal of biological chemistry. 1983;258(9):5392–9. 6222053

[37] Demaurex N, Distelhorst C. Cell biology. Apoptosis–the calcium connection. Science (New York, NY). 2003;300(5616):65–7. 10.1126/science.1083628 12677047

[38] Paasch U, Grunewald S, Dathe S, Glander HJ. Mitochondria of human spermatozoa are preferentially susceptible to apoptosis. Annals of the New York Academy of Sciences. 2004;1030:403–409. 10.1196/annals.1329.050 15659823

[39] Bender CE, Fitzgerald P, Tait SWG, Llambi F, McStay GP, Tupper DO, et al Mitochondrial pathway of apoptosis is ancestral in metazoans. Proceedings of the National Academy of Sciences of the United States of America. 2012;109(13):4904–9. 10.1073/pnas.1120680109 22416118 PMC3324028

[40] Belardetti F, Schacher S, Siegelbaum SA. Action potentials, macroscopic and single channel currents recorded from growth cones of Aplysia neurones in culture. The Journal of physiology. 1986;374:289–313. 10.1113/jphysiol.1986.sp016080 2427703 PMC1182721

[41] Crest M, Gola M. Large conductance Ca(2+)-activated K+ channels are involved in both spike shaping and firing regulation in Helix neurones. The Journal of physiology. 1993;465:265–87. 10.1113/jphysiol.1993.sp019676 8229836 PMC1175429

[42] Araque A, Buño W. Fast BK-type channel mediates the Ca(2+)-activated K(+) current in crayfish muscle. Journal of neurophysiology. 1999;82(4):1655–1661. 10.1152/jn.1999.82.4.1655 10515956

[43] Latorre R, Miller C. Conduction and selectivity in potassium channels. The Journal of Membrane Biology. 1983;71(1-2):11–30. 10.1007/bf01870671 6300405

[44] Guerrero A, Espinal J, Wood CD, Rendón JM, Carneiro J, Martínez-Mekler G, et al Niflumic acid disrupts marine spermatozoan chemotaxis without impairing the spatiotemporal detection of chemoattractant gradients. Journal of cell science. 2013;126(6):1477–87. 10.1242/jcs.121442 23418354

[45] Chung JJ, Shim SH, Everley Ra, Gygi SP, Zhuang X, Clapham DE. Structurally distinct Ca2+ signaling domains of sperm flagella orchestrate tyrosine phosphorylation and motility. Cell. 2014;157(4):808–822. 10.1016/j.cell.2014.02.056 24813608 PMC4032590

[46] Miller MR, Kenny SJ, Mannowetz N, Mansell SA, Wojcik M, Mendoza S, et al Asymmetrically Positioned Flagellar Control Units Regulate Human Sperm Rotation. Cell Reports. 2018;24(10):2606–2613. 10.1016/j.celrep.2018.08.016 30184496 PMC6177234

[47] HODGKIN AL, HUXLEY AF. A quantitative description of membrane current and its application to conduction and excitation in nerve. The Journal of physiology. 1952;117(4):500–44. 10.1113/jphysiol.1952.sp004764 12991237 PMC1392413

[48] Bönigk W, Loogen A, Seifert R, Kashikar N, Klemm C, Krause E, et al An atypical CNG channel activated by a single cGMP molecule controls sperm chemotaxis. Science signaling. 2009;2(94):ra68 10.1126/scisignal.2000516 19861689

[49] Nishigaki T, Darszon A. Real-time measurements of the interactions between fluorescent speract and its sperm receptor. Developmental biology. 2000;223(1):17–26. 10.1006/dbio.2000.9734 10864457

[50] Nishigaki T, Zamudio FZ, Possani LD, Darszon A. Time-resolved sperm responses to an egg peptide measured by stopped-flow fluorometry. Biochemical and biophysical research communications. 2001;284(2):531–535. 10.1006/bbrc.2001.5000 11394914

[51] Bentley JK, Garbers DL. Receptor-mediated responses of plasma membranes isolated from Lytechinus pictus spermatozoa. Biology of reproduction. 1986;35(5):1249–59. 10.1095/biolreprod35.5.1249 2881584

[52] Ward GE, Moy GW, Vacquier VD. Phosphorylation of membrane-bound guanylate cyclase of sea urchin spermatozoa. The Journal of cell biology. 1986;103(1):95–101. 10.1083/jcb.103.1.95 2873144 PMC2113804

[53] Ramarao CS, Garbers DL. Purification and properties of the phosphorylated form of guanylate cyclase. The Journal of biological chemistry. 1988;263(3):1524–9. 2891712

[54] Ruiz-Herrero T, Estrada J, Guantes R, Miguez DG. A Tunable Coarse-Grained Model for Ligand-Receptor Interaction. PLoS Computational Biology. 2013;9(11):e1003274 10.1371/journal.pcbi.1003274 24244115 PMC3828130

[55] Dangott LJ, Garbers DL. Identification and partial characterization of the receptor for speract. The Journal of biological chemistry. 1984;259(22):13712–6. 6094527

[56] Bentley JK, Tubb DJ, Garbers DL, Garberssgll DL. Receptor-mediated activation of spermatozoan guanylate cyclase. The Journal of biological chemistry. 1986;261(32):14859–62. 2876990

[57] Shimomura H, Dangott LJ, Garbers DL, Shimomurass H, Dangottsb LJ, Garberssqt DL, et al Covalent coupling of a resact analogue to guanylate cyclase. The Journal of biological chemistry. 1986;261(33):15778–82. 2877982

[58] Singh S, Lowe DG, Thorpe DS, Rodriguez H, Kuang WJ, Dangott LJ, et al Membrane guanylate cyclase is a cell-surface receptor with homology to protein kinases. Nature. 1988;334(6184):708–12. 10.1038/334708a0 2901039

[59] Galindo BBE, de la Vega-Beltrán J, de la Vega-Beltrán JL, Labarca P, Vacquier VD, Darszon A. Sp-tetraKCNG: A novel cyclic nucleotide gated K+ channel. Biochemical and …. 2007;354(3):668–675. 10.1016/j.bbrc.2007.01.035 17254550

[60] Cukkemane A, Seifert R, Kaupp UB. Cooperative and uncooperative cyclic-nucleotide-gated ion channels. Trends in biochemical sciences. 2011;36(1):55–64. 10.1016/j.tibs.2010.07.004 20729090

[61] Labarca P, Santi C, Zapata O, Morales E, Beltr’an C, Li’evano a, et al A cAMP regulated K+-selective channel from the sea urchin sperm plasma membrane. Developmental biology. 1996;174(2):271–80. 10.1006/dbio.1996.0072 8631499

[62] Gauss R, Seifert R, Kaupp UB. Molecular identification of a hyperpolarization-activated channel in sea urchin sperm. Nature. 1998;393(6685):583–7. 10.1038/31248 9634235

[63] Sánchez D, Labarca P, Darszon A, Sa D. Sea urchin sperm cation-selective channels directly modulated by cAMP. FEBS letters. 2001;503(1):111–5. 10.1016/s0014-5793(01)02713-2 11513865

[64] Shin KS, Maertens C, Proenza C, Rothberg BS, Yellen G. Inactivation in HCN channels results from reclosure of the activation gate: desensitization to voltage. Neuron. 2004;41(5):737–44. 10.1016/s0896-6273(04)00083-2 15003173

[65] Nomura M, Vacquier VD. Proteins associated with soluble adenylyl cyclase in sea urchin sperm flagella. Cell motility and the cytoskeleton. 2006;63(9):582–90. 10.1002/cm.20147 16847896

[66] Lee HC. A membrane potential-sensitive Na+-H+ exchange system in flagella isolated from sea urchin spermatozoa. The Journal of biological chemistry. 1984;259(24):15315–9. 6096367

[67] Lee HC. Sodium and proton transport in flagella isolated from sea urchin spermatozoa. The Journal of biological chemistry. 1984;259(8):4957–63. 6325412

[68] Reynaud E, De de La Torre L, Zapata O, Liévano a, Darszon A. Ionic bases of the membrane potential and intracellular pH changes induced by speract in swollen sea urchin sperm. FEBS letters. 1993;329(1-2):210–4. 10.1016/0014-5793(93)80223-h 8354397

[69] Lee HC, Garbers DL. Modulation of the voltage-sensitive Na+/H+ exchange in sea urchin spermatozoa through membrane potential changes induced by the egg peptide speract. The Journal of biological chemistry. 1986;261(34):16026–32. 2430965

[70] Dash RK, Beard Da. Analysis of cardiac mitochondrial Na+-Ca2+ exchanger kinetics with a biophysical model of mitochondrial Ca2+ handling suggests a 3:1 stoichiometry. The Journal of physiology. 2008;586(13):3267–85. 10.1113/jphysiol.2008.151977 18467367 PMC2538784

[71] Cai X, Clapham DE. Evolutionary genomics reveals lineage-specific gene loss and rapid evolution of a sperm-specific ion channel complex: CatSpers and CatSperbeta. PloS one. 2008;3(10):e3569 10.1371/journal.pone.0003569 18974790 PMC2572835

[72] Kirichok Y, Navarro B, Clapham DE. Whole-cell patch-clamp measurements of spermatozoa reveal an alkaline-activated Ca2+ channel. Nature. 2006;439(7077):737–40. 10.1038/nature04417 16467839

[73] Lishko PV, Botchkina IL, Kirichok Y. Progesterone activates the principal Ca2+ channel of human sperm. Nature. 2011;471(7338):387–91. 10.1038/nature09767 21412339

[74] Strünker T, Goodwin N, Brenker C, Kashikar ND, Weyand I, Seifert R, et al The CatSper channel mediates progesterone-induced Ca2+ influx in human sperm. Nature. 2011;471(7338):382–6. 10.1038/nature09769 21412338

[75] Soetaert K, Petzoldt T, Setzer RW. Solving Differential Equations in R: Package deSolve. Journal of Statistical Software. 2010;33(9). 10.18637/jss.v033.i09

[76] Shin KS, Rothberg BS, Yellen G. Blocker state dependence and trapping in hyperpolarization-activated cation channels: evidence for an intracellular activation gate. The Journal of general physiology. 2001;117(2):91–101. 10.1085/jgp.117.2.91 11158163 PMC2217248

[77] Talavera K, Nilius B. Biophysics and structure-function relationship of T-type Ca2+ channels. Cell Calcium. 2006;40(2):97–114. 10.1016/j.ceca.2006.04.013 16777221

[78] Guerrero A, Nishigaki T, Carneiro J, Yoshiro T, Wood CD, Darszon A. Tuning sperm chemotaxis by calcium burst timing. Developmental Biology. 2010;344(1):52–65. 10.1016/j.ydbio.2010.04.013 20435032

[79] Lee HC, Johnson C, Epel D. Changes in internal pH associated with initiation of motility and acrosome reaction of sea urchin sperm. Developmental biology. 1983;95(1):31–45. 10.1016/0012-1606(83)90004-0 6825930

[80] Babcock DF, Bosma MM, Battaglia DE, Darszon A. Early persistent activation of sperm K+ channels by the egg peptide speract. Proceedings of the National Academy of Sciences of the United States of America. 1992;89(13):6001–5. 10.1073/pnas.89.13.6001 1631086 PMC402126

[81] Rodríguez E, Darszon A. Intracellular sodium changes during the speract response and the acrosome reaction in sea urchin sperm. The Journal of physiology. 2003;546(Pt 1):89–100. 10.1113/jphysiol.2002.030510 12509481 PMC2342476

[82] Balke CW, Rose WC, Marban E, Wier WG. Macroscopic and unitary properties of physiological ion flux through T-type Ca2+ channels in guinea-pig heart cells. The Journal of Physiology. 1992;456(1):247–265. 10.1113/jphysiol.1992.sp019335 1338097 PMC1175680

[83] Nishigaki T, González-cota AL. Pathologies of Calcium Channels. Weiss N, Koschak A, editors. Berlin, Heidelberg: Springer Berlin Heidelberg; 2014 Available from: http://link.springer.com/10.1007/978-3-642-40282-1.

[84] González-Martínez MT, Guerrero a, Morales E, de De La Torre L, Darszon a. A depolarization can trigger Ca2+ uptake and the acrosome reaction when preceded by a hyperpolarization in L. pictus sea urchin sperm. Developmental biology. 1992;150(1):193–202. 10.1016/0012-1606(92)90018-c 1371478

[85] Beltrán C, Zapata O, Darszon a. Membrane potential regulates sea urchin sperm adenylylcyclase. Biochemistry. 1996;35(23):7591–8. 10.1021/bi952806v 8652541

[86] Hille B. Ion Channels of Excitable Membranes (3rd Edition). 3rd ed Sinauer Associates Inc 2001-07; 2001.

[87] Cavanaugh GM. Formulae and Methods VI of the Marine Biological Laboratory Chemical Room. Woodshole, Massachusetts: Marine Biological Laboratory; 1956.

[88] Bentley JK, Shimomura H, Garbers DL. Retention of a functional resact receptor in isolated sperm plasma membranes. Cell. 1986;45(2):281–288. 10.1016/0092-8674(86)90392-2 2870813

