# Supplementary figures and images for: Modular analysis of the control of flagellar Ca2+-spike trains produced by CatSper and CaV channels in sea urchin sperm
Source: PLoS Comput Biol. 2020 Mar 2;16(3):e1007605. doi: 10.1371/journal.pcbi.1007605 (PMC7067495; doi:10.1371/journal.pcbi.1007605)

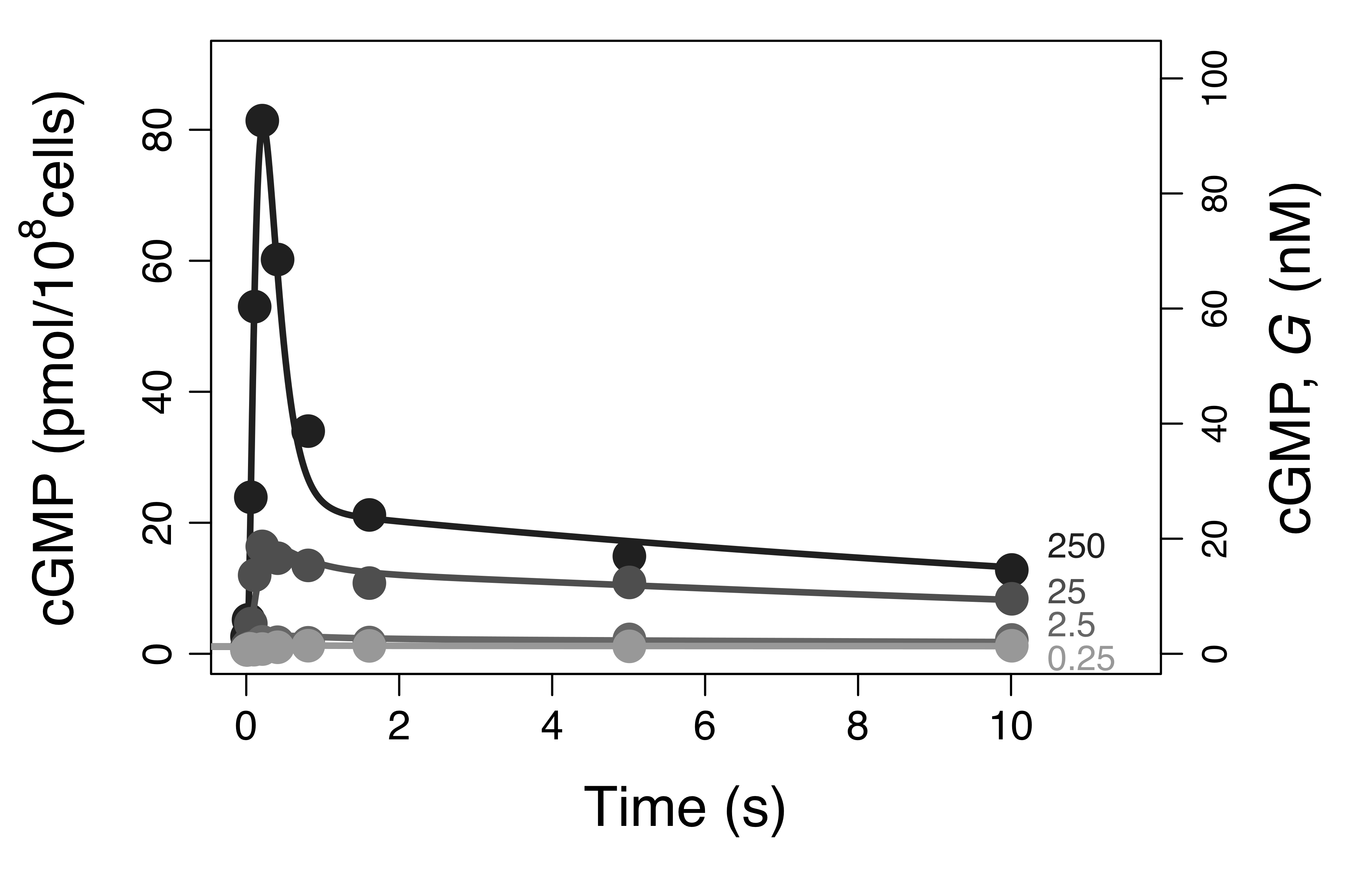

Supplement: S1 Fig — The graph shows the time courses of cGMP concentration elicited by the indicated SAP concentrations (values in nM). For each SAP concentration (encoded in grey shades) the dots represent experimental values read from the figures in [22] and the lines are the single cell numerical solutions of the variable G in the Upstream module, with state space {S, RF, RH, RL, G} that best fit the ensemble of the data (i.e. the four curves simultaneously). The values of RT, r2, r3, δG, kL and kH were fitted while fixing the remaining parameters (Table 2). Because the measurements of the SAP-receptor association rate constant r1 are technically more reliable than those of the number of receptors RT (reported values include 14000 [88, 22], 3 × 10^5^ [28] and 1 × 10^6^ [27] molecules per cell), we fixed the value of r1 and fitted RT. We fixed also the value of the factor that converts the amount receptor-bound SAP molecules to soluble concentration θR = s/NA = 6.14 × 10^−5^ nMcell based on the reported cell density s = 3.7 × 10^7^ cellmL−1 [22]. Finally, Gr was preset to 1.24 nM [22] and the condition σG = Gr/δG held during the fitting. (TIF) [file pcbi.1007605.s001.tif]

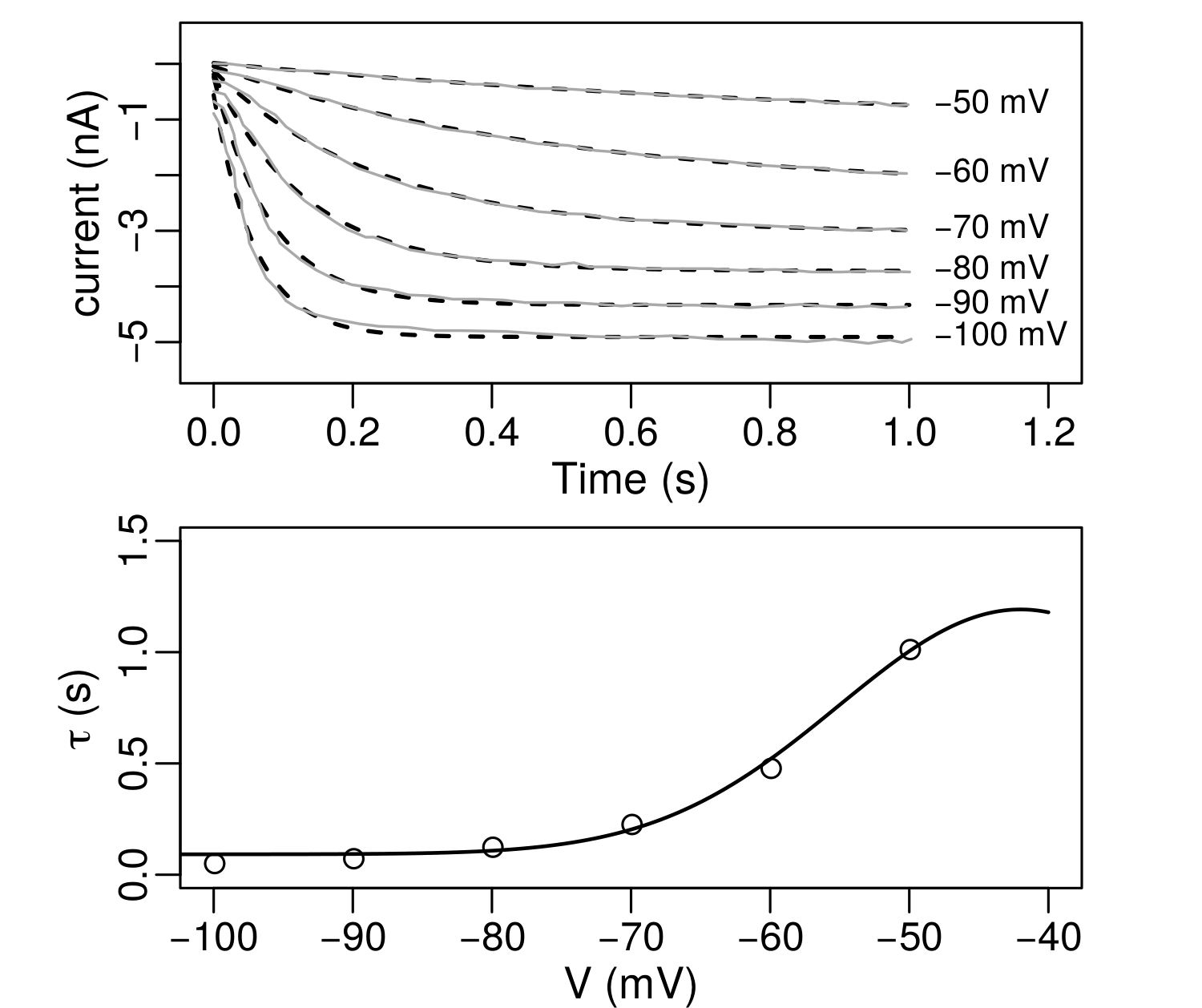

Supplement: S2 Fig — In the upper panel, experimental traces of ionic currents measured by whole cell patch clamp technique in HEK cells expressing heterologously spHCN and loaded with photoactivatable cAMP analog, which in turn was uncaged by UV light. The set of currents correspond to different voltage pulses (indicated at the end of the trace). Data extracted from figure 4a of [62] (gray lines). Each trace was fitted to an an exponential function (black dashed lines) in order to estimate the characteristic activation time (τ). In the lower pannel, the set of estimated characteristic times was fitted to a Gaussian function, which is our proposed form for the voltage-dependent characteristic time of spHCN gating (Eqs 20 and 21). (TIF) [file pcbi.1007605.s002.tif]

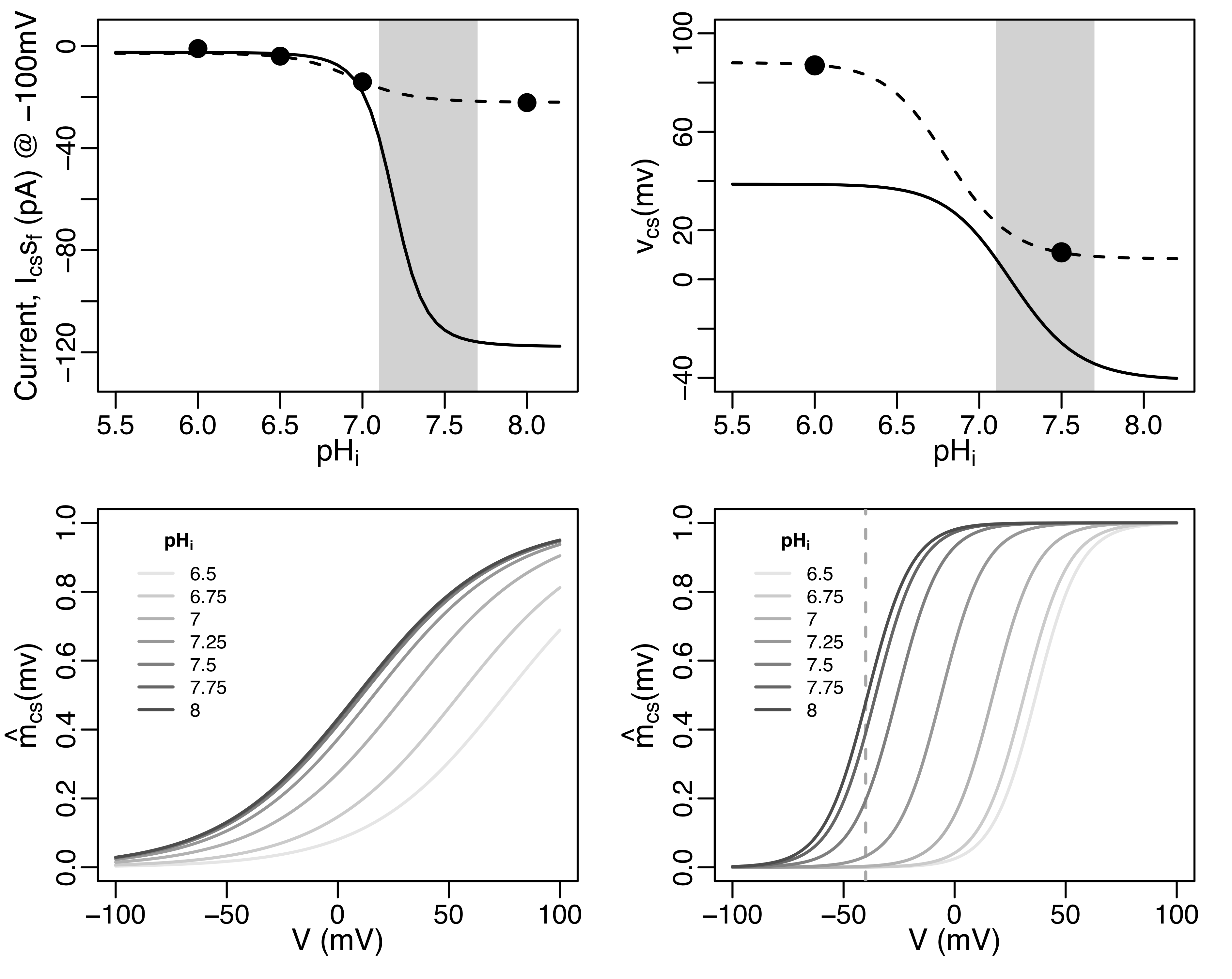

Supplement: S3 Fig — In A and B, experimental data on mouse sperm (circles) are shown, along with model fittings (lines) related to the voltage- and pHi-dependent gate variable in equilibrium, m^cs. Model fittings in mouse and sea urchin cases are plotted with dashed and bold lines, respectively. The experimental data of A correspond to the current amplitude of divalent ions (symmetrical Ba2+) produced by taking the holding voltage from 0 mV to −100 mV, under different pHi values, and measured by whole-cell patch-clamp in mouse sperm, (data extracted from Fig. 4c of [72]). Mouse data displayed in A and B were simultaneously fitted to the Eqs 44 and 40. To calibrate sea urchin’s m^cs, we took the parameters obtained with mouse data as a starting point and manually adjusted them, constraining that the pHi sensitivity should be within the physiological pHi response observed in sea urchin sperm (area marked in gray). C and D correspond to the G/V (conductance/voltage) curves of mouse and sea urchin CatSper, respectively, using the Eq 39. In C, the parameters reported in [72] were used). In panel D, the resting membrane potential is indicated with a gray dashed line as reference. Taking into account that the S4 segment of CatSper voltage sensor domain has more positive charges in the sea urchin homologue protein that in the mammalian counterparts [12], we envisioned that its voltage sensitivity should be steeper. Thus, a 3-fold decrease was introduced to the voltage sensitivity parameter, s2, as an initial guess. This last parameter has not been estimated in sea urchin due to the lack of patch-clamp measurements. (TIF) [file pcbi.1007605.s003.tif]

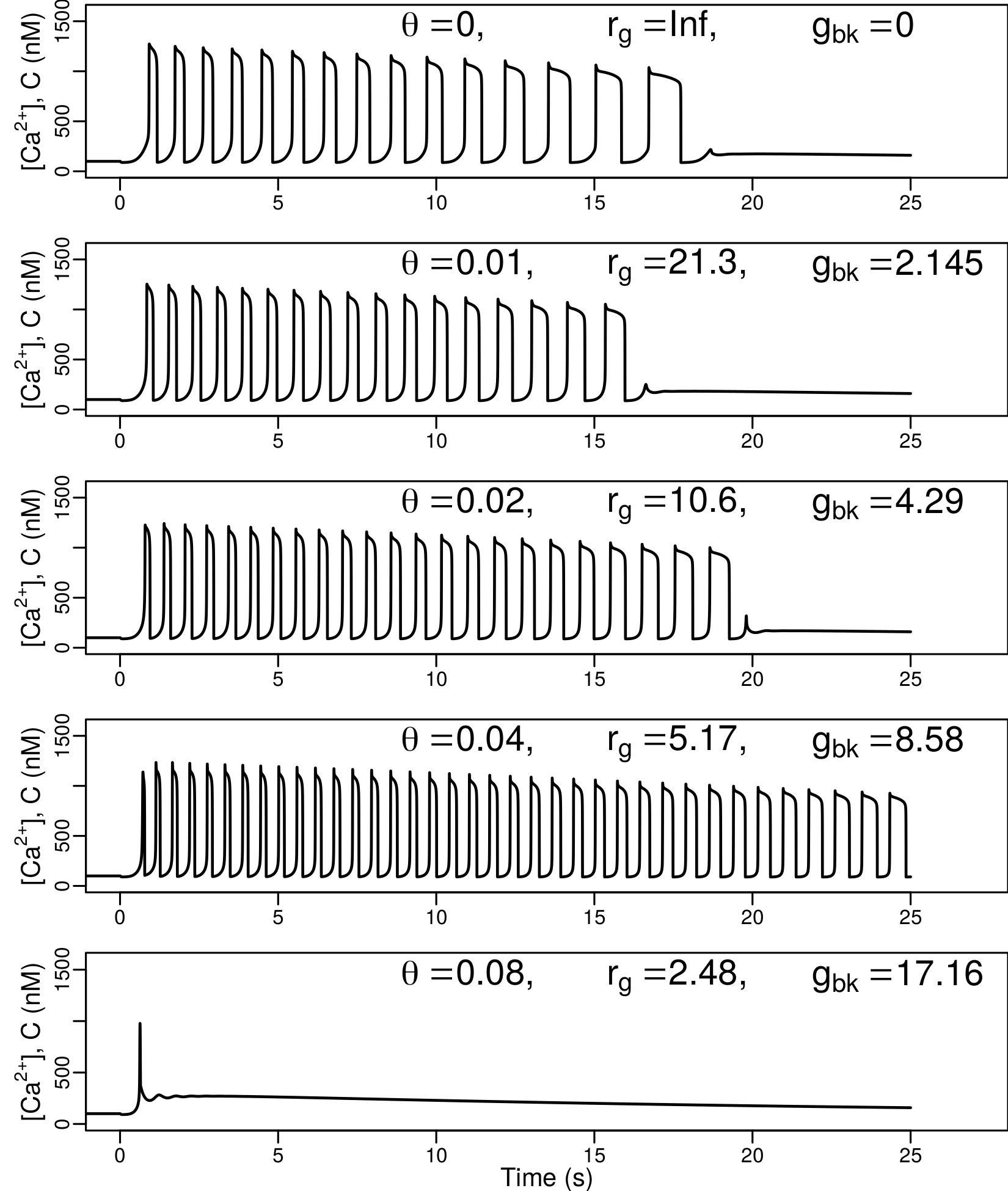

Supplement: S4 Fig — Starting from the model that includes the CatSper module, numerical solutions for calcium are shown under different values of the weighting parameter θ, which controls the percentage of module CaV + BK that is being added. The parameters are set according to Eqs 48–52 with θcv = θ and θcs = (1 − θ). For reference, the original CatSper-only scenario is shown first, i.e. θ = 0, and the subsequent rows correspond to the gradual increase of θ. The effect of this parameter on the channel densities is reported in the coefficient rg, which measures the total conductance ratio CatSper/CaV, as well as the corresponding gbk value. The greater the value of rg, the greater the predominance of CatSper on the total calcium conductance with respect to CaV. (TIFF) [file pcbi.1007605.s004.tiff]

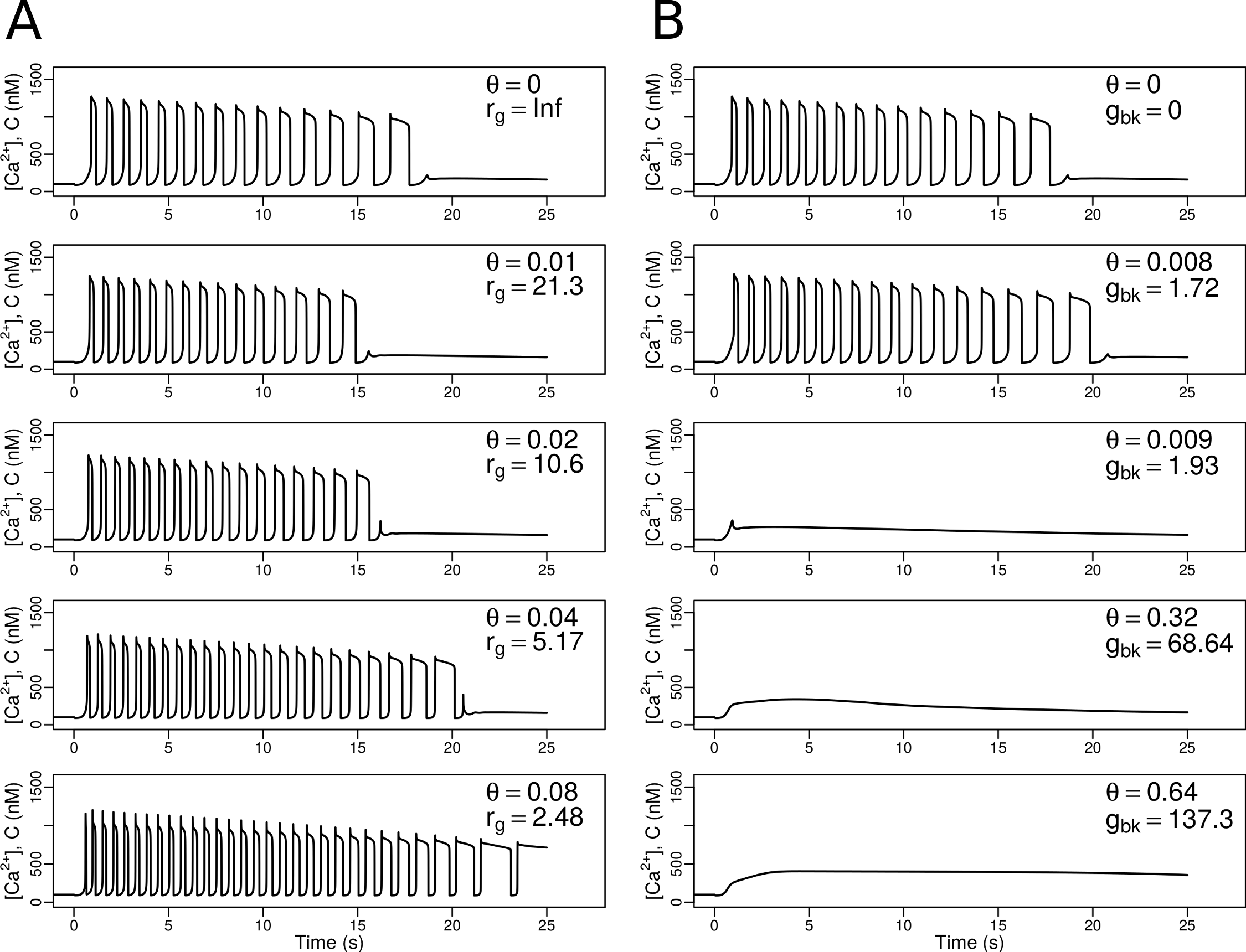

Supplement: S5 Fig — In A and B, we show numerical solutions for calcium under different values of the weighting parameter θ, with either BK or CaV conductance density set to 0, respectively. As reference, the original scenario of only CatSper, i.e. θ = 0, is shown in the top row, while the subsequent rows correspond to the gradual increase of θ. In A, the reference value of the CaV conductance density, gcv, is multiplied directly by θ, whereas the reference value of CatSper conductance density is multiplied by (1—θ); The resulting ratio of CatSper/CaV conductance densities, rg, is shown for each θ value. In B, θ sets the fraction of the reference value of gbk by multiplying the conductance density of BK; unlike scenario A, the conductance density of CatSper is not weighted by θ. For each titration, the corresponding modified gbk value is shown. (TIFF) [file pcbi.1007605.s005.tiff]
