# Supplementary material for: Modular analysis of the control of flagellar Ca2+-spike trains produced by CatSper and CaV channels in sea urchin sperm
Source: PLoS Comput Biol. 2020 Mar 2;16(3):e1007605. doi: 10.1371/journal.pcbi.1007605 (PMC7067495; doi:10.1371/journal.pcbi.1007605)
Supplement: S1 Algorithm — (PDF) [file pcbi.1007605.s006.pdf]

Algorithm Two-phase parameter space exploration

```
with: $\overrightarrow{\lambda}^{cv}$, $\overrightarrow{\lambda}^{cs}$

$s \leftarrow 0.04$
$n \leftarrow 500$
```

Phase 1

```
$L \leftarrow \{\}$
for each $\theta^{cv} \in \{0.0001, 0.1, 0.2, \ldots, 2\}$ do
  for each $\theta^{cs} \in \{0.0001, 0.1, 0.2, \ldots, 2\}$ do
    $\overrightarrow{\lambda} \leftarrow \theta^{cv}\overrightarrow{\lambda}^{cv} + \theta^{cs}\overrightarrow{\lambda}^{cs}$
    $L \leftarrow L \cup \{\overrightarrow{\lambda}\}$
  end for
end for

while No coalescence of clouds of points in the projection plane $(g_{cv}, g_{cs})$ do
  $\overrightarrow{\lambda} \leftarrow$ Sample($L$)
  for each $\lambda_i \in \overrightarrow{\lambda}$ do
    if $\lambda_i \notin$ Upstream Module Parameter Subset then
      $x \sim Normal(0, s)$
      $\epsilon \leftarrow e^x$
    else
      $\epsilon \leftarrow 1$
    end if
    $\lambda_i \leftarrow \lambda_i \times \epsilon$
  end for
  $L \leftarrow L \cup \{\overrightarrow{\lambda}\}$
end while
```

Phase 2

```
$L_{Sel} \leftarrow \{\}$
$L_{Uns} \leftarrow \{\}$
for each $\overrightarrow{\lambda} \in L$ do
  if $\overrightarrow{\lambda}$ is Coherent with Observed Data (Sec.  4.4) then
    $L_{Sel} \leftarrow L_{Sel} \cup \{\lambda\}$
  end if
end for

while No stable clustering in projection plane $(g_{cv}, g_{cs})$ do
  $iter \leftarrow 0$
  repeat
    increment $iter$
    $\overrightarrow{\lambda} \leftarrow$ Sample($L_{Sel}$)
    for each $\lambda_i \in \overrightarrow{\lambda}$ do
      if $\lambda_i \notin$ Upstream Module Parameter Subset then
        $x \sim Normal(0, s)$
        $\epsilon \leftarrow e^x$
      else
        $\epsilon \leftarrow 1$
      end if
      $\lambda_i \leftarrow \lambda_i \times \epsilon$
    end for
    if $\overrightarrow{\lambda}$ is Coherent with Observed Data (Sec.  4.4) then
      $L_{Sel} \leftarrow L_{Sel} \cup \{\overrightarrow{\lambda}\}$
    else
      $L_{Uns} \leftarrow L_{Uns} \cup \{\overrightarrow{\lambda}\}$
    end if
  until $iter = n$
end while
```
